# The characteristics and spatiotemporal evolution of heatwaves and droughts across six typical regions in China

**DOI:** 10.1038/s41598-026-43650-1

**Published:** 2026-03-18

**Authors:** Ya Yang, Dongdong Liu

**Affiliations:** College of Resource and Environmental Engineering, Key Laboratory of Karst Georesources and Environment, Ministry of Education, Guizhou University, Guiyang, 550025 China

**Keywords:** Meteorological drought, Hydrological drought, Hydrological processes, Typical regions, Spatiotemporal dynamics, Climate sciences, Environmental sciences, Hydrology, Natural hazards, Water resources

## Abstract

Extreme heatwaves and droughts pose critical threats to global water, food, and socioeconomic security. However, comprehensive understanding of their spatiotemporal dynamics, underlying drivers, and cascading impacts remains limited, particularly across China’s ecologically vulnerable regions. This study employs GLDAS data, validated against NOAA flux tower observations (R^2^ = 0.97), to analyze heatwave and drought characteristics across six major Chinese basins (2004–2023). Marked increases are observed in frequency (0.019 vs. 0.022 events/yr), intensity (0.012 °C vs. 0.14 mm/yr), duration (0.039 vs. 0.4 days/yr), and earlier onset (0.23 vs. 0.045 days/yr). Under the persistence of current trends, our extrapolation suggests that by 2050, these metrics are projected to increase 1.14–1.33-fold, with onset advancing by 1.22–4.33 days, driven predominantly by anthropogenic forcing. Spatially, the Yangtze Basin exhibits high frequency, the Qinghai–Tibet Plateau high intensity, the Songliao Basin moderate frequency with high intensity, and the Loess Plateau and Pearl Basin frequent shorter events. Northern regions experience persistent warming, while the southeast shows increased precipitation, evapotranspiration, and groundwater and soil moisture. Key correlations include temperature with heatwave intensity, runoff with duration, and food production with heatwave attributes. Drought correlates strongly with precipitation, groundwater, terrestrial water storage, and population growth. Mitigating greenhouse gas emissions is imperative to reduce escalating disruptions across hydrological, agricultural, and societal systems.

**Supplementary Information:**

The online version contains supplementary material available at 10.1038/s41598-026-43650-1.

## Introduction

Global warming has intensified the impacts of extreme climate events on society and the environment, with widespread occurrences in regions such as Europe^1^, Central Asia^2^, France^3^, the U.S^4^., Australia^5^, and China^6–8^. Heatwaves and droughts, as representative extreme events, pose severe threats to human health^9,10^, ecosystem stability^11,12^, infrastructure^13^, economic and social development^14,15^, agricultural security^16^, and natural resource availability^2^. Between 1951 and 2021, China’s annual surface temperature increased at a rate of 0.26 °C per decade, significantly exceeding the global rate of 0.15 °C per decade^17^, exacerbating economic and environmental pressures.

Heatwaves, defined by prolonged periods of extreme high temperatures^2,18,19^, are characterized by their intensity, frequency, and duration. These events have been well-documented and are becoming more severe^1,3,16^. For example, the 2003 European heatwave resulted in over 70,000 deaths and substantial agricultural losses^4^. In 2013, China’s heatwave affected more than 500 million people across nine provinces^20^. Similarly, in 2018, a record-breaking heatwave on the Korean Peninsula led to 4508 heat-related illnesses and 48 fatalities^21^.

Sustained high temperatures significantly impact the hydrological cycle^4^, affecting precipitation, surface runoff, evapotranspiration, root zone soil moisture (RZSM), ground water storage (GWS), and terrestrial water storage (TWS). Trenberth et al.^22^ highlighted those heatwaves influence global precipitation patterns, alter river flow regimes^23,24^, increase evaporation rates^25,26^, and deplete RZSM and GWS^1,5^. These changes, in turn, have cascading effects on energy demand^27^, social stability^28^, natural disasters^29^, and ecosystem resilience^30^. Climate model projections indicate that these trends will persist and intensify throughout the twenty-first century^31^.

Heatwave identification typically uses either absolute or relative threshold methods^32^. The absolute method, based on fixed temperature values^33^, varies regionally,for instance, the China Meteorological Administration sets the threshold at 35°C, while the World Meteorological Organization uses 32°C. To enhance regional applicability, the percentile threshold method has become more widely used^4,16^. Heatwaves are often associated with meteorological drought, driven by high temperatures, insufficient rainfall, and excessive evapotranspiration^26^. When heat extends to soil, soil moisture drought can occur, a process known as drought propagation^34,35^. Huang et al.^36^ and Wu et al.^37^ have investigated the response time and thresholds for the transition from meteorological to hydrological drought. Zhang et al.^38^ highlighted a significant correlation between propagation time and human activities, while Lin et al.^26^ noted that a time lag exists between meteorological and hydrological droughts, with variations in intensity across different regions. Previous studies have focused on the general characteristics of these events, such as intensity, frequency, and duration, but spatial propagation is often overlooked (Table 1). Heatwave and drought characteristics evolve over time, with intensity centroids shifting due to climate and human factors^48^. Therefore, incorporating spatial propagation is vital for effective climate adaptation policies.

**Table 1:** Previous studies on the influences of heatwave events on the hydrological processes.

Location	Period	Hydrological variable						Characteristics of heatwave events					Characteristics of drought events					References
		P	SR	ET	GWS	RZSM	TWS	Num	Dur	Int	Ear	Ori	Num	Dur	Int	Ear	Ori	
Global	2010–2100	×	×	×	×	√	√	√	√	√	√	√	√	√	√	×	×	Yin et al^19^.
Global	1979–2018	×	×	×	×	×	×	√	√	√	×	√	×	×	×	×	×	Lo et al^39^.
Global	1979–2018	×	×	√	√	×	×	×	√	√	×	×	×	×	×	×	×	Wang et al^18^.
Central Asia	1981–2020	×	×	×	×	√	×	√	√	√	×	×	×	×	×	×	×	Wang et al^2^.
European	1996–2014	×	×	√	×	√	×	×	×	×	√	×	×	×	×	×	×	Lansu et al^1^.
French Alps	2000–2016	√	×	√	×	×	×	×	×	√	×	×	×	×	×	×	×	Corona-Lozada et al^3^.
USA	2003–2022	√	√	√	√	√	√	√	√	√	×	×	×	×	×	×	×	Hao et al^4^.
Australian	1911–2012	×	×	×	×	√	×	√	√	√	√	×	×	×	×	×	×	Perkins et al^5^.
China	1981–2005	√	×	×	×	×	×	×	√	√	×	×	×	×	×	×	×	You and Wang^8^.
China	1961–2018	×	√	×	×	×	×	√	√	×	×	×	×	×	×	×	×	Chen et al^6^.
China	1960–2018	×	×	×	×	×	×	√	×	√	×	×	×	×	×	×	×	Wang and Yan^7^.
CLP	1961–2100	×	×	×	×	×	×	√	√	√	×	×	×	×	×	×	×	Si et al^40^.
CLP	1960–2016	√	×	×	×	×	×	√	√	×	×	×	√	√	×	×	×	Sun et al^41^.
QTP	1978–2017	×	×	×	×	×	×	√	√	√	×	×	×	×	×	×	×	Zhang and Yao^42^,
QTP	2001–2020	×	×	×	×	×	×	×	√	√	×	×	×	×	×	×	×	Chen et al^43^.
QTP	1980–2018	√		√	×	×	×	×	√	√	×	√	×	√	√	×	√	Lin et al^26^.
NCP	1995–2014 2041–2100	×	×	×	×	×	×	×	√	√	×	×	×	×	×	×	×	Zhang et al^44^.
NCP	2023	×	×	×	×	×	×	×	×	√	√	×	×	×	×	×	×	Xiao et al^45^.
YR	2022	√	×	×	×	√	×	×	×	√	×	×	×	×	√	×	×	Chen et al^23^.
YR	2022	√	×	√	×	√	×	×	×	√	×	×	×	×	√	×	×	Chen et al^24^.
PRB	1960–2100	√	√	√	×	√	×	√	×	√	×	×	√	×	×	×	×	Li et al^25^.
PRB	1961–2020	√	×	×	×	×	×	×	√	√	×	×	×	√	√	×	×	Liu et al^46^.
SRB	1961–2018	√	×	×	×	×	×	×	×	×	×	×	×	√	√	×	×	Gong^47^,
China (NCP; PRB; CLP; QTP; YR; SRB)	2004–2050	√	√	√	√	√	√	√	√	√	√	√	√	√	√	√	√	This study

P: precipitation; SR: surface runoff; ET: evapotranspiration; GWS: ground water storage; RZSM: root zone soil moisture; TWS: Terrestrial water storage; Num: number; Dur: duration; Int: intensity; Ear: Earliest day; Ori: orientation.

NCP: North China Plain; PRB: Pearl River Basin; CLP: Loess Plateau; QTP: Qinghai-Tibetan Plateau; YR: Yangtze River Basin; SRB: Songliao River Basin.

China’s vulnerability to heatwaves and droughts is heightened by its complex geology, large population, agricultural sector, and monsoon climate^6,7^. Research across various regions highlights significant spatiotemporal changes in heatwave and drought events. From 1960–2016, the Loess Plateau saw an increase in such events, with central droughts and southeastern heatwaves^40,41^. In the Qinghai-Tibet Plateau (2000–2021), heatwave frequency and intensity varied significantly, linked to climate change^26,42,43^. North China (1979–2021) experienced more frequent heatwaves and droughts, with strong spatial–temporal correlations^44,45^. In the Yangtze River Basin (1961–2020), both frequency and intensity of these events increased, showing synergistic variations^23,24^. The Pearl River Basin (1960–2015) experienced heatwaves primarily in the central and northern regions^25,46^.

Despite global and regional assessments increasingly link extreme events to anthropogenic forcing (Table 1), there is still a lack of integrated analyses in China that jointly examine: (i) long-term trends in heatwave and drought characteristics; (ii) their spatial propagation across major basins; and (iii) their statistical associations with greenhouse gas concentrations. Bridging this gap is essential for connecting observed changes in hydroclimatic extremes to broader climate forcing pathways and for providing policy-relevant information for mitigation and adaptation. To address these gaps, this study develops a unified framework to investigate the spatial–temporal dynamics of meteorological and hydrological droughts, together with heatwaves, across six typical regions of China that are representative in terms of resources, agriculture, economy, and ecology. Using GLDAS data validated against in-situ flux tower observations, we (1) consistently identify heatwave and RZSM-based drought events and quantify their key characteristics (number, duration, intensity, and earliest onset) from 2004 to 2023; (2) analyze their spatial patterns and propagation by tracking intensity centroid migration in each basin; (3) assess the responses of meteorological and hydrological variables to heatwave occurrence; and (4) explore potential drivers by relating event characteristics to climatic, hydrological, socioeconomic indicators and greenhouse gas concentrations. By providing a basin-comparative, process-oriented, and driver-informed perspective, our study aims to advance the understanding of coupled heatwave–drought dynamics in China and to offer a more robust scientific basis for water resources management and climate adaptation planning.

## Data and methodology

### Study area

To analyze spatial–temporal dynamics, six representative regions across China were selected, each significant in at least one domain: resources, agriculture, industry, economy, or ecology. The Loess Plateau (CLP, 33°34′–41°49′N, 101°53′–114°30′E), an arid to semi-arid region prone to severe soil erosion and water scarcity, supports over 8.5% of China’s population and is dominated by a continental monsoon climate with highly variable precipitation^9,49,50^. The North China Plain (NCP, 36.5°–42.5°N, 113.5°–119.5°E), a densely populated agricultural and industrial hub accounting for one-fifth of the national population, faces increasing heatwaves and droughts under global warming, threatening its ecological and socio-economic stability^44,45^. The Qinghai-Tibet Plateau (QTP, 25°59′–40°29′N, 73°27′–104°30′E), known as the “Water Tower of Asia,” is a high-altitude region experiencing rapid warming (0.033°C/yr) and serves as a critical area for climate change studies with minimal direct human disturbance^42,51,52^. The Pearl River Basin (PRB, 102°14′–115°53′E, 21°31′–26°49′N), China’s third-largest river basin, has a humid subtropical climate but suffers from growing water stress and rising drought frequency due to rapid urbanization^25,53^. The Yangtze River Basin (YR, 24°30′–35°45′N, 90°33′–122°25′E) sustains over 400 million people and contributes more than 40% of national GDP, characterized by strong precipitation seasonality and complex terrain-induced variability^54,55^. The Songliao River Basin (SRB, 115°32′–135°06′E, 38°43′–53°43′N), a key industrial-agricultural region in Northeast China with a continental monsoon climate, experiences wide precipitation and temperature gradients, and is increasingly impacted by extreme rainfall events^56^,Cheng et al., 2023).

### Datasets

This study utilizes multiple datasets, including the GLDAS^57^ Noah Land Surface Model L4 (GLDAS_NOAH025_3H, V2.1) with 3-hourly intervals and a 0.25° × 0.25° resolution, the GLDAS Catchment Land Surface Model L4 (GLDAS_CLSM025_DA1_D, V2.2) with daily intervals and the same resolution, and in-situ flux tower data from the National Centers for Environmental Information (NCEI) under the NOAA, USA. The GLDAS_NOAH025_3H dataset was employed to extract 3-hourly climate variables, including temperature, precipitation, and relative humidity, while the GLDAS_CLSM025_DA1_D dataset provided daily hydrological fluxes such as evapotranspiration, GWS, RZSM, and TWS.

**Fig. 1:**
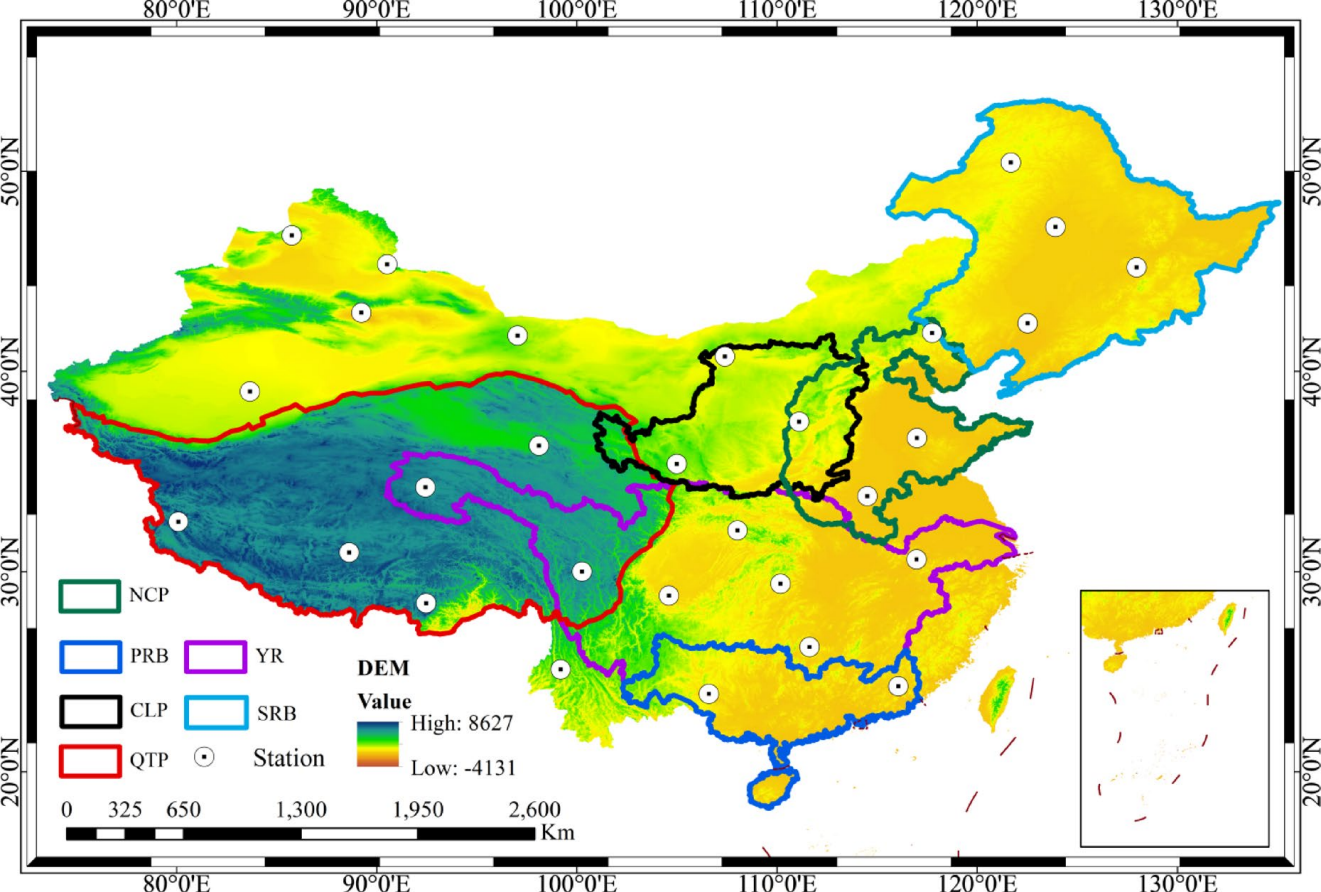
Overview of the location of the study area. NCP: North China Plain; PRB: Pearl River Basin; CLP: Loess Plateau; QTP: Qinghai-Tibetan Plateau; YR: Yangtze River Basin; SRB: Songliao River Basin. QTP, CLP, and NCP are topographic/geomorphological zones, while YR, PRB, and SRB are hydrological basin zones. The intersecting and overlapping boundaries within the map reflect the rational superposition of topographic units and basin units within the natural geographic pattern. Elaborated with the ArcGIS 10.2 software, https://desktop.arcgis.com/zh-cn/arcmap/.

To ensure data quality, in-situ flux tower observations from NOAA were used to validate the relationship between GLDAS simulations and observed temperature (Fig. S1) and rainfall (Fig. S2). High-quality, ground-based temperature observations served as a benchmark for assessing GLDAS data accuracy, following the validation approach of Hao et al.^4^. The selected in-situ data sites, spanning 2004–2023, were approximately evenly distributed across the six study regions to ensure broad applicability (Fig. 1). A detailed overview of the flux tower data is presented in Table 2. Additionally, data from the World Meteorological Organization’s Greenhouse Gas Bulletin were incorporated to analyze the drivers of heatwaves and droughts, specifically annual average concentrations of carbon dioxide (CO₂), atmospheric methane (CH₄), and nitrous oxide (N₂O) from 2004 to 2023. Table 3 provides a comprehensive summary of the data sources utilized in this study. This study employs two complementary approaches: (1) spatially explicit mapping to reveal heterogeneous patterns and local hotspots, and (2) regional averaging to provide integrated indicators for cross-regional comparison and overall trend quantification. The regional means should therefore be interpreted as aggregate signals, while detailed spatial distributions and probability analyses capture within-region variability.

**Table 2:** A summary of the station data used in this study to validate data availability.

ID	Station Name	Latitude	Longitude	Variable	Period
1	LINGLING	26.2333333	111.6166666	t2m, tp	2004–2023
2	MEI XIAN	24.2833333	116.0666666	t2m, tp	2004–2023
3	SHIQUANHE	32.5	80.0833333	t2m, tp	2004–2023
4	XIHUA	33.7833333	114.5166666	t2m, tp	2004–2023
5	SANGZHI	29.4	110.1666666	t2m, tp	2004–2023
6	TUOTUOHE	34.2166666	92.4333333	t2m, tp	2004–2023
7	JINAN/TSINAN	36.6833333	116.9833333	t2m, tp	2004–2023
8	BAISE	23.9	106.6	t2m, tp	2004–2023
9	YIBIN	28.8	104.6	t2m, tp	2004–2023
10	ANQING	30.6166666	116.9666667	t2m, tp	2004–2023
11	LHUNZE	28.4166666	92.4666666	t2m, tp	2004–2023
12	ZHANGWU	42.4166666	122.5333333	t2m, tp	2004–2023
13	LITANG	30	100.2666666	t2m, tp	2004–2023
14	WEICHANG	41.9333333	117.75	t2m, tp	2004–2023
15	WANYUAN	32.0666666	108.0333333	t2m, tp	2004–2023
16	BAOSHAN	25.1166666	99.1833333	t2m, tp	2004–2023
17	HUAJIALING	35.3833333	105	t2m, tp	2004–2023
18	LISHI	37.5	111.1	t2m, tp	2004–2023
19	TAZHONG	39	83.6666666	t2m, tp	2004–2023
20	MAZONG SHAN	41.8	97.0333333	t2m, tp	2004–2023
21	SHANGZHI	45.2166666	127.9666666	t2m, tp	2004–2023
22	TULIHE	50.45	121.7	t2m, tp	2004–2023
23	LINHE	40.7666666	107.4	t2m, tp	2004–2023
24	TURPAN	42.95	89.2297222	t2m, tp	2004–2023
25	DOULAN	36.3	98.1	t2m, tp	2004–2023
26	KARAMAY	45.3666666	90.5333333	t2m, tp	2004–2023
27	HOBOKSAR	46.8166666	85.75	t2m, tp	2004–2023
28	SANJIAZI	47.239628	123.918131	t2m, tp	2004–2023
29	XAINZA	30.95	88.6333333	t2m, tp	2004–2023

t2m: temperature at 2 m; tp: precipitation.

**Table 3:** The characteristics of data sources and details used in this study.

Categories	Derived variables	Units	Data resource
Topography	Elevation	m	https://earthengine.google.com
Meteorology (satellite)	Temperature	℃	https://disc.gsfc.nasa.gov/datasets/GLDAS_NOAH025_3H_2.1/summary
	Precipitation	mm	
	Relative humidity	%	
Meteorology (station)	Temperature	℃	https://www.ncei.noaa.gov/data/global-summary-of-the-day/archive/
	Precipitation	mm	
Hydrology (satellite)	Surface runoff	mm	https://disc.gsfc.nasa.gov/datasets/GLDAS_CLSM025_DA1_D_2.2/summary
	Evapotranspiration	mm	
	Ground Water Storage	mm	
	Root Zone Soil Moisture	kg/m^2^	
	Terrestrial Water Storage	mm	
Sociology	Net Population Growth	people	https://www.gov.cn/
	Total Food Production	kg	
	Gross Domestic Product	RMB	
Greenhouse gas	Carbon dioxide	ppm	https://wmo.int/publication-series/wmo-greenhouse-gas-bulletin-no-20
	Atmospheric methane	pbb	
	Nitrous oxide	pbb	

### Methodology

#### Heatwave and drought event identification

**Fig. 2:**
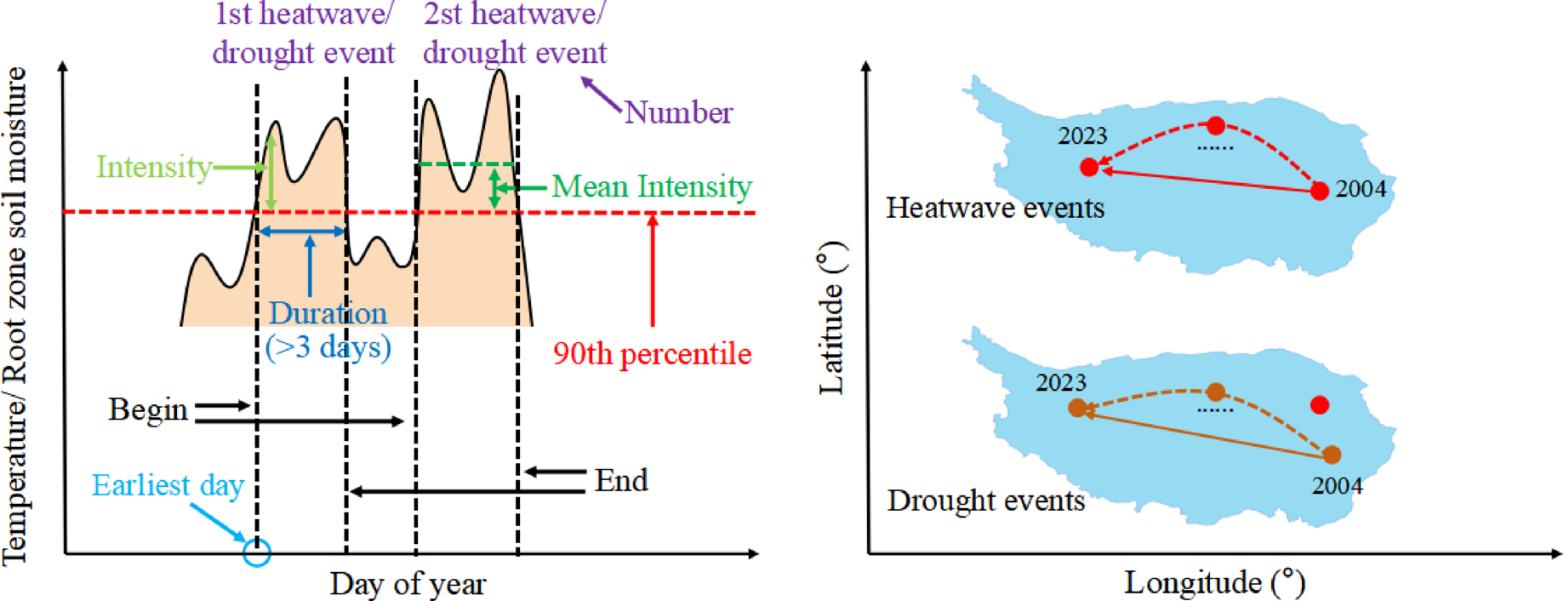
Schematic definition of temporal and spatial characteristics of heatwave and drought events, including number, duration, intensity, earliest day of first event, and the propagation trajectory of the center of mass of the event between 2004 and 2023. Note that heatwave and drought events do not correspond to each other in space and time, just to offer a schematic of the definitions.

To evaluate the impact of extreme heat on hydrological processes, heatwave events were first identified using daily mean temperature data from GLDAS. Following the relative definition method of Ha et al.^16^, Wang et al.^2^, Hao et al.^4^, You and Wang^8^., Chen et al.^6^, all of which adopt the 90th percentile as the threshold for heatwave identification, a heatwave was defined as a period during the warm season (May–October) when the daily mean temperature exceeded the 90th percentile of the warm-season temperature distribution. This aligns with the standard practice used in the cited references and ensures consistency in extreme event characterization. Heatwave events were then identified as periods of at least three consecutive days surpassing this threshold. Heatwave events primarily reflect meteorological conditions driven by excessive temperatures^26^. When prolonged high temperatures coincide with insufficient soil moisture, soil moisture drought may develop. However, meteorological drought and soil moisture drought do not exhibit a strict one-to-one correspondence,their occurrence can be subject to temporal and spatial lags and may be modulated by local human activities. Drought events were identified analogously to heatwaves using a relative threshold approach, but with RZSM in the 0–40 cm layer as the variable. This layer was selected because it directly reflects agricultural and ecological drought^26,58^, responds sensitively to meteorological forcing^4^, and avoids strong near-surface noise or deep groundwater interference. A drought event was defined when daily RZSM fell below the 10th percentile of its warm-season distribution for at least three consecutive days. The 10th percentile corresponds to statistically extreme dry conditions^59,60^ and a critical threshold for vegetation drought response^61,62^, ensuring symmetric and comparable identification with heatwaves. Key characteristics of heatwave and drought events include their number, duration, intensity, earliest onset, and propagation (Fig. 2). The number of events was determined by counting occurrences meeting the respective criteria throughout the study period. Duration was measured as the number of days from the event’s onset to its termination, with a minimum duration of three days, given that an event is only recognized if the threshold is exceeded for at least three consecutive days. Intensity was quantified based on the extent to which the defining metric (temperature for heatwaves, RZSM for droughts) deviated from its threshold. The earliest day of an event was identified as the first occurrence of conditions meeting the heatwave or drought criteria within a given year. To quantify the spatial propagation of heatwave and drought events and obtain the decadal-scale migration trajectory, we calculated the intensity-weighted centroid for each event, computed the annual mean centroid by averaging the centroids of all events occurring each year from 2004 to 2023, connected these 20 annual-mean points chronologically to form the propagation path, and applied linear regression to the time series of annual-mean centroid longitudes and latitudes to determine the overall direction and rate of movement. The centroid longitude (${\mathrm{C}}_{{{\mathrm{lon}}}}$) and latitude (${\mathrm{C}}_{{{\mathrm{lat}}}}$) were computed as:

1 $${\mathrm{C}}_{{{\mathrm{lon}}}} { = }\frac{{\mathop \sum \nolimits_{{\text{i = 1}}}^{{\mathrm{N}}} {\mathrm{I}}_{{\mathrm{i}}} \cdot {\mathrm{lon}}_{{\mathrm{i}}} }}{{\mathop \sum \nolimits_{{\text{i = 1}}}^{{\mathrm{N}}} {\mathrm{I}}_{{\mathrm{i}}} }}{\text{, C}}_{{{\mathrm{lat}}}} { = }\frac{{\mathop \sum \nolimits_{{\text{i = 1}}}^{{\mathrm{N}}} {\mathrm{I}}_{{\mathrm{i}}} \cdot {\mathrm{lat}}_{{\mathrm{i}}} }}{{\mathop \sum \nolimits_{{\text{i = 1}}}^{{\mathrm{N}}} {\mathrm{I}}_{{\mathrm{i}}} }}$$

where ${\mathrm{I}}_{{\mathrm{i}}}$ is the intensity at grid cell ${\mathrm{i}}$, and (${\mathrm{lon}}_{{\mathrm{i}}}$, ${\mathrm{lat}}_{{\mathrm{i}}}$) are its coordinates. This centroid represents the “center of mass” of the event’s intensity field.

#### Meteorological and hydrological response assessment

To study the immediate to short-term responses of meteorological (i.e., precipitation, temperature) and hydrological variables (i.e., evapotranspiration, surface runoff, TWS, GWS, RZSM) to heatwave events, we analyzed the differences in hydrological factors between periods immediately preceding and following each event. Following the approach of Hao et al.^4^, a three-day buffer interval was adopted to capture changes occurring proximate to the heatwave. Specifically, the average value of each variable over the three days before the heatwave onset was subtracted from its average over the three days after the heatwave termination. This short-term window is designed to isolate rapid hydrological reactions—such as increased surface runoff, enhanced evapotranspiration, and depletion of near-surface soil moisture—that are directly forced by extreme heat on daily timescales. It should be noted, however, that slower-responding components like GWS and total TWS are influenced by integrated climate forcing, seasonal recharge, and anthropogenic withdrawals over weeks to months. Consequently, the changes observed in GWS/TWS within this three-day frame likely reflect coupling with faster surface processes or short-term fluctuations rather than the full, lagged response of these deeper, slower reservoirs.

#### Driver factor analysis and trend projection

First, trends in the characteristics of heatwave and drought events from 2004 to 2023 were analyzed based on the identification results in Section "Spatial–temporal distribution of hydrological variables". Using these statistically significant historical trends, we constructed empirical extrapolations for the period 2024–2050 to depict potential future changes in heatwave and drought characteristics under the assumption that recent trends persist. It is crucial to note that these extrapolations are not equivalent to physically-based climate model projections. Instead, they serve as scenario-based references that illustrate the possible magnitude and direction of changes in the coming decades if the observed forcing and responses continue along their recent trajectory. These references are intended to inform preliminary risk assessment and long-term planning. Additionally, correlations between heatwave and drought characteristics and climatic, hydrological, and societal factors were examined to elucidate the mechanisms driving the formation and evolution of these extreme events. Understanding these relationships enhances the accuracy of predictions regarding changes in their frequency and intensity. Finally, greenhouse gas concentration data from the World Meteorological Organization were analyzed to explore potential links between heatwave and drought events and CO₂, CH₄, and N₂O levels. This analysis aimed to identify possible causal relationships between gas emissions and extreme climate events and to explore potential mitigation pathways.

### Statistical analysis

Regression analyses were conducted to assess temporal changes in heatwave and drought characteristics, their correlations with climatic, hydrological, and societal factors, and their associations with greenhouse gases (CO₂, CH₄, and N₂O). Statistical significance was determined at P < 0.01 and P < 0.05. The coefficient of determination (R^2^) was used to evaluate the explanatory power of the regression models. Additionally, a 95% confidence band and a 95% prediction band were employed to indicate the reliability of the regression model and its projections. Visualization and spatial analysis were performed using ArcMap (V10.2), Origin (V2021), and Python (V3.9) to generate maps and figures.

## Results

### Spatial–temporal distribution of hydrological variables

**Fig. 3:**
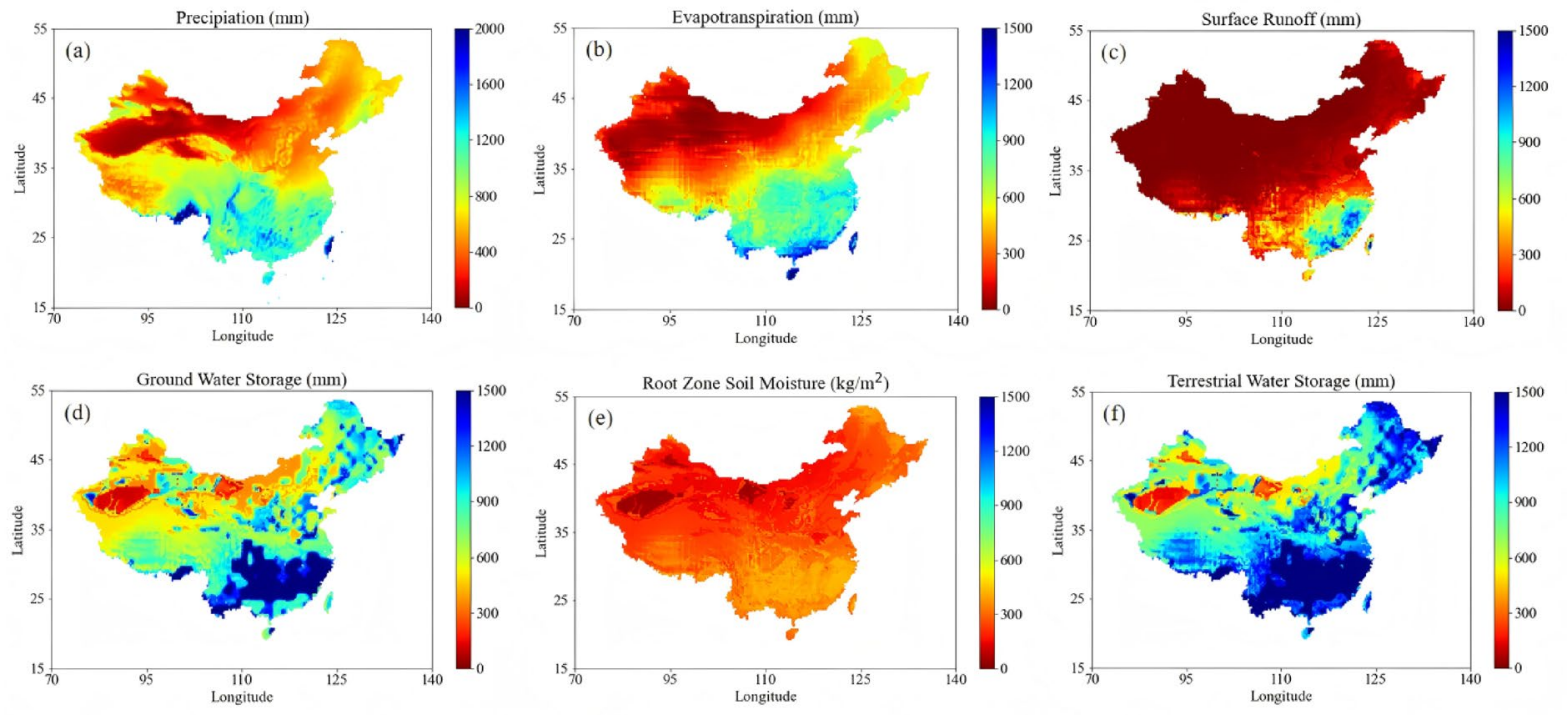
Spatial distribution of annual (**a**) Precipitation, (**b**) Evapotranspiration, (**c**) Surface Runoff, (**d**) Ground Water Storage, (**e**) Root Zone Soil Moisture, and (**f**) Terrestrial Water Storage in the China (2004–2023). Elaborated with Spyder (Anaconda3) based on Python 3.9, https://www.spyder-ide.org/, https://www.python.org/

Figure 3 illustrates the spatial distributions of China’s mean annual hydrological variables from 2004 to 2023. Precipitation exhibits a pronounced southeast-to-northwest gradient (Fig. 3a), exceeding 1000 mm/yr in the humid southeast and declining below 400 mm/yr in the arid northwest. Evapotranspiration is strongly moisture-coupled (Fig. 3b), peaking at 1200 mm/yr in vegetated southeastern regions, while remaining water-limited (300–600 mm/yr) in the northwest despite high atmospheric demand. The Qinghai–Tibet Plateau exhibits thermally suppressed evapotranspiration. Surface runoff (Fig. 3c) is highly heterogeneous, exceeding 900 mm/yr in southeastern basins due to high rainfall and infiltration, but falling below 300 mm/yr in northwestern arid regions. Groundwater storage (Fig. 3d) mirrors runoff patterns, with high recharge (~ 1500 mm) in the southeast and limited storage (300–600 mm) in the northwest. Root zone soil moisture (Fig. 3e) shows parallel contrasts, maximized in southeastern coastal and northeastern black soil zones (900–1200 kg/m^2^), moderated by irrigation in the North China Plain, and minimized (≤ 600 kg/m^2^) in northwestern and Tibetan cryospheric regions due to moisture deficits. Total water storage (Fig. 3f) integrates these patterns, reflecting abundant hydrological cycling in the southeast (> 1,200 mm) and acute water stress in the northwest, exacerbated by anthropogenic extraction and climate shifts.

### Spatial–temporal propagation of meteorological drought events

**Fig. 4:**
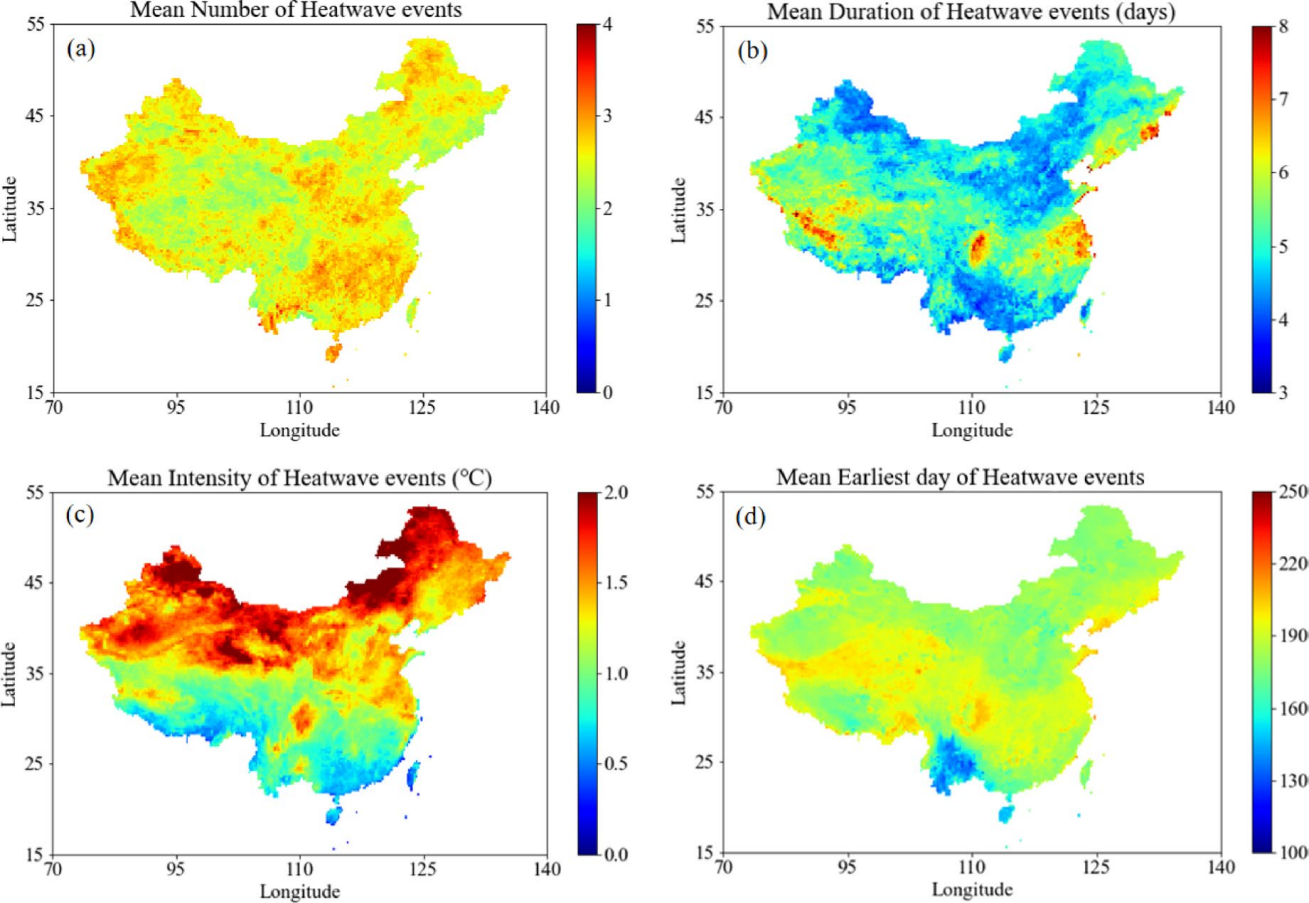
Spatial coverage of (**a**) total number of heatwave events, (**b**) mean duration of heatwave events, (**c**) mean intensity of heatwave events, and (**d**) mean earliest day of the year for heatwave events in the China (2004–2023). Elaborated with Spyder (Anaconda3) based on Python 3.9, https://www.spyder-ide.org/, https://www.python.org/.

Figure 4 depicts spatiotemporal patterns of heatwave characteristics across China from 2004 to 2023. Southeastern regions experience the highest frequency (~ 55 events), while the QTP and Xinjiang show the lowest. Duration exceeds 6 days in South China and the YR, but averages only 3–4 days in northern and northwestern areas. Thermal intensity is most pronounced in northern China (> 2.0 °C), moderate in the YR (1.0–1.5 °C), and weakest in the PRB and QTP (0.5–1.0 °C). The earliest onset occurs in April–May in South China, progressing to July–August in northeastern regions, with the QTP exhibiting high interannual variability.

**Fig. 5:**
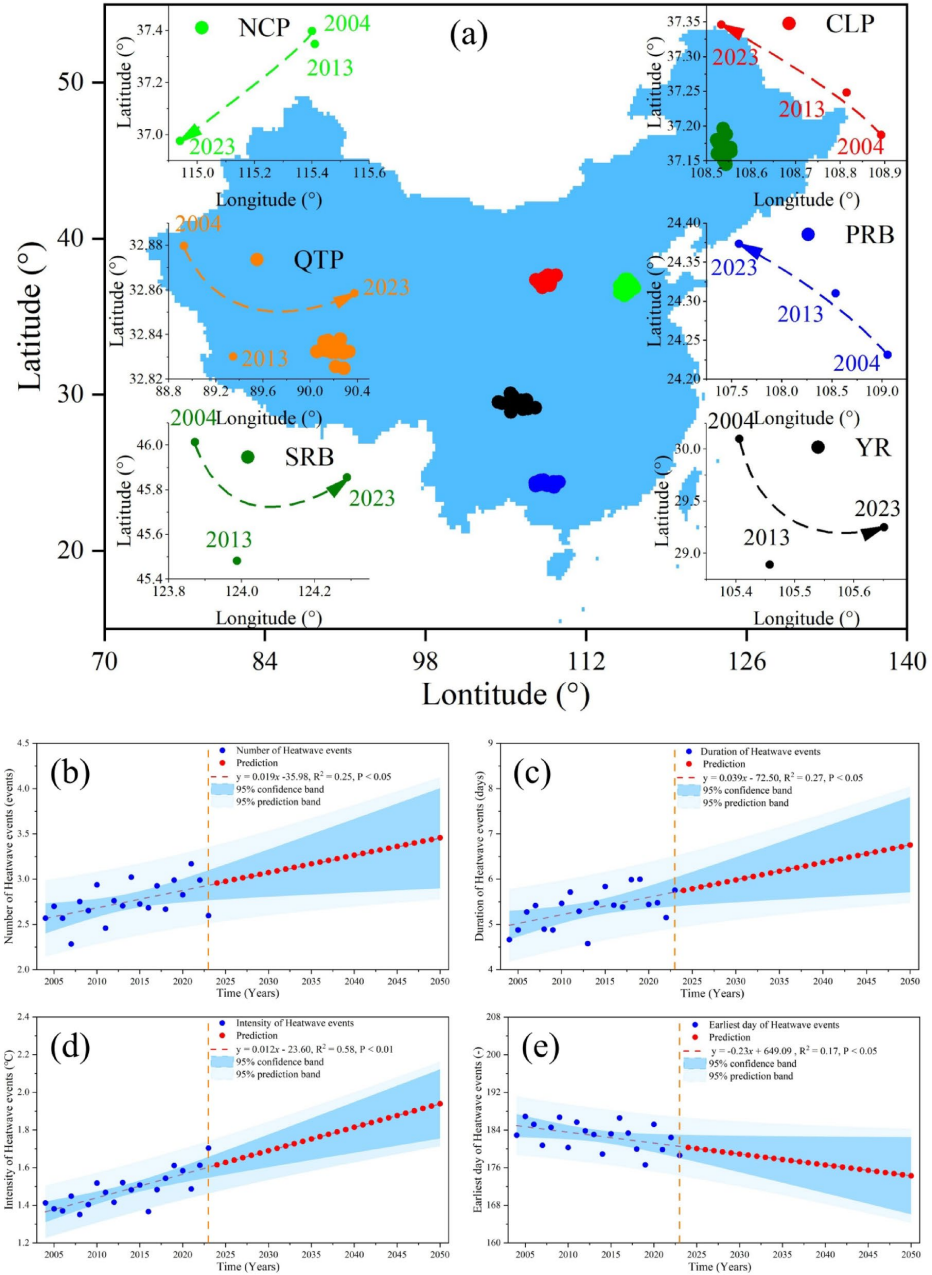
The changes of migration trajectories (**a**), mean number (**b**), mean duration (**c**), mean intensity (**d**), and mean earliest day of the year (**e**) of drought events in six typical regions of China during 2004–2023, and the projection of 2024–2050. Elaborated with Spyder (Anaconda3) based on Python 3.9, supported by Matplotlib & Basema, https://www.spyder-ide.org/, https://www.python.org/, https://matplotlib.org/basemap/.

Figure 5 presents migration trajectories and multi-decadal trends. Intensity centroids display divergent shifts: southwestward in the NCP, inward over the QTP, and northwestward on the Loess Plateau. All metrics show statistically significant increases (P < 0.05): frequency rising at 0.019 events/yr, duration at 0.039 days/yr, intensity at 0.012°C/yr (P < 0.01), and onset advancing by 0.23 days/yr. Projections suggest by 2050 a 133.13% increase in frequency, a one-day longer duration, 13.81% greater intensity, and an onset 4.33 days earlier.

**Fig. 6:**
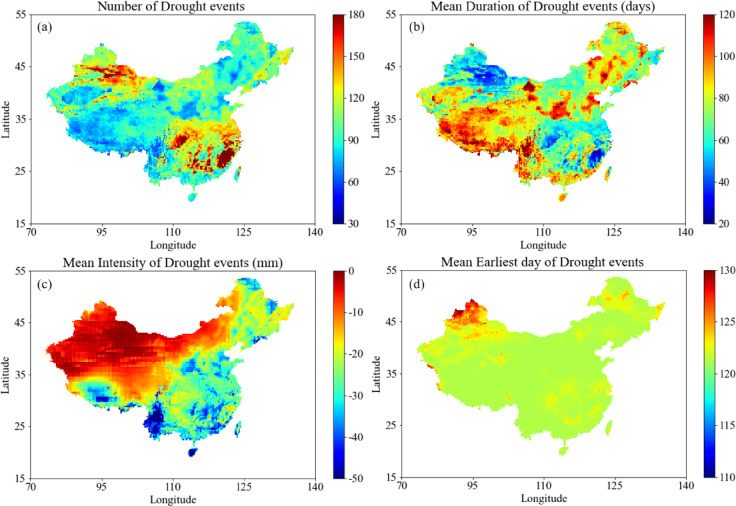
Probability distributions and magnitude statistics of total number of heatwave events (**a** vs. **b**), mean duration of heatwave events (**c** vs. **d**), mean intensity of heatwave events (**e** vs. **f**), and mean earliest day of the year for heatwave events (**g** vs. **h**) in six typical regions of China (2004–2023).

Figure 6 illustrates probability distributions across six regions. The Loess Plateau and YR show the highest event counts (40–60), while the QTP remains lowest with a left-skewed distribution. Duration ranges from 3–8 days, shortest in the NCP and PRB, and longest in the QTP and YR. Intensity is highest in the Songhua River Basin (peaking at 1.0–3.0°C) and lowest in the PRB. The PRB and YR exhibit earlier skewed onsets, whereas the QTP commences around day 200.

### Spatial–temporal propagation of hydrological drought events

**Fig. 7:**
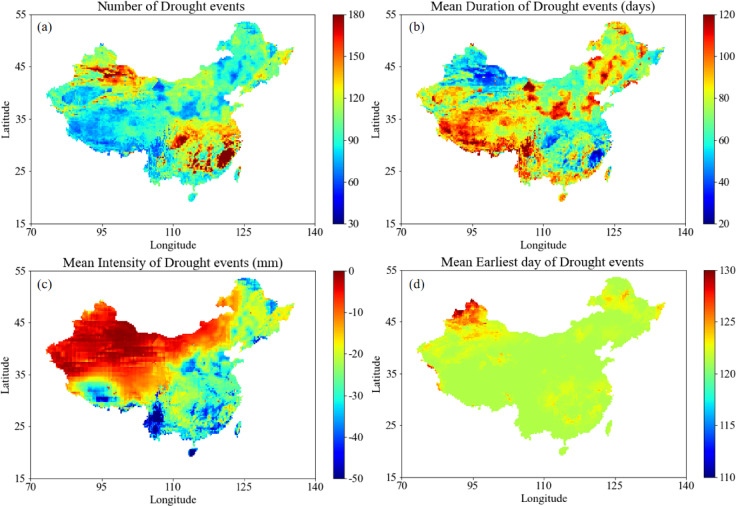
Spatial coverage of (**a**) total number of drought events, (**b**) mean duration of drought events, (**c**) mean intensity of drought events, and (**d**) mean earliest day of the year for drought events in the China (2004–2023). Elaborated with Spyder (Anaconda3) based on Python 3.9, https://www.spyder-ide.org/, https://www.python.org/.

Figure 7 presents the characteristics of drought events in China from 2004 to 2023, including number, duration, intensity, and earliest occurrence. The number of drought events (Fig. 7a) is highest in eastern China, particularly in the NCP and middle-lower YR (> 150 events), influenced by high population density and complex precipitation patterns. The west, including the QTP and parts of Xinjiang, experiences fewer events. Duration (Fig. 7b) is longest in the northwest and west, especially the SRB and QTP (> 90 days), while shorter durations (< 40 days) are observed in parts of Xinjiang and East China. Intensity (Fig. 7c) is highest in the south and southeast (e.g., PRB, YR, NCP), exceeding -40 mm in some areas, while the west and northwest (e.g., QTP, Xinjiang, Gansu, Inner Mongolia) exhibit lower values (-10 ~ 0 mm). Earliest occurrence (Fig. 7d) follows a south-to-north delay, starting February–March in South China, April–May in the NCP, and May–June in the Northeast. In the west, the pattern is more complex, with droughts beginning after May–June in the QTP and after April–May in northwestern regions like Xinjiang.

**Fig. 8:**
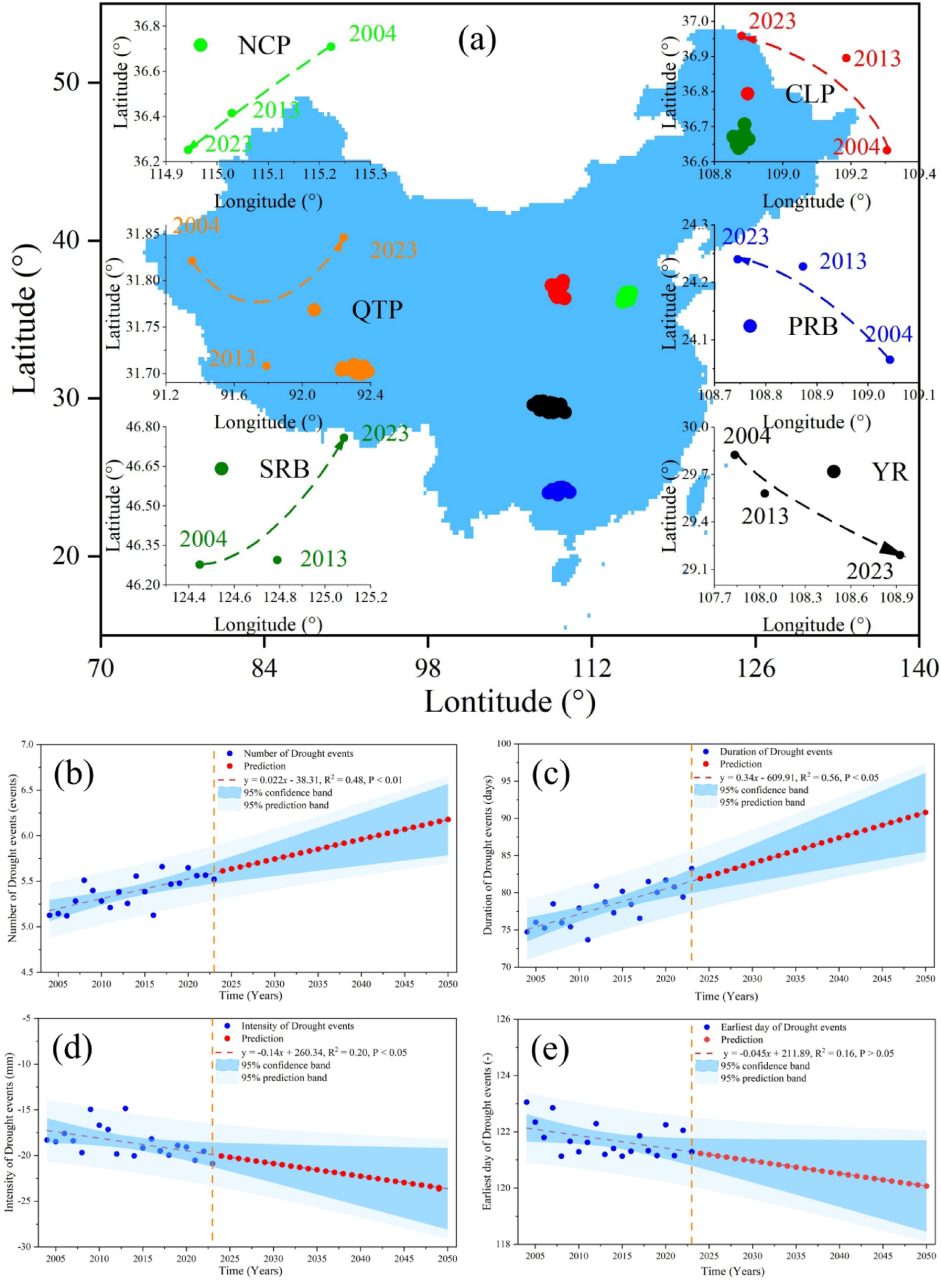
The changes of migration trajectories (**a**), mean number (**b**), mean duration (**c**), mean intensity (**d**), and mean earliest day of the year (**e**) of heatwave events in six typical regions of China during 2004–2023, and the projection of 2024–2050. Elaborated with Spyder (Anaconda3) based on Python 3.9, supported by Matplotlib & Basema, https://www.spyder-ide.org/, https://www.python.org/, https://matplotlib.org/basemap/.

Figure 8 illustrates drought migration and trends from 2004 to 2023, with projections for 2024–2050. Figure 8a shows intensity center shifts: southwestward in the NCP, expanding outward in the QTP, northeastward in the SRB, northwestward in the CLP, diffusing northwestward in the PRB, and spreading southeastward in the YR. Drought number (Fig. 8b) increased significantly (P < 0.01; 0.022 events/yr.), projected to reach 111.92% of 2023 levels by 2050. Duration (Fig. 8c) increased (P < 0.05; 0.034 days/yr.), extending 7.54 days longer by 2050. Intensity (Fig. 8d) rose (P < 0.05; 0.14 mm/yr.), projected to be 13.23% higher by 2050. The earliest day (Fig. 8e) advanced at 0.045 days/yr., though not significantly, suggesting the first drought event in 2050 will occur 1.22 days earlier than in 2023.

**Fig. 9:**
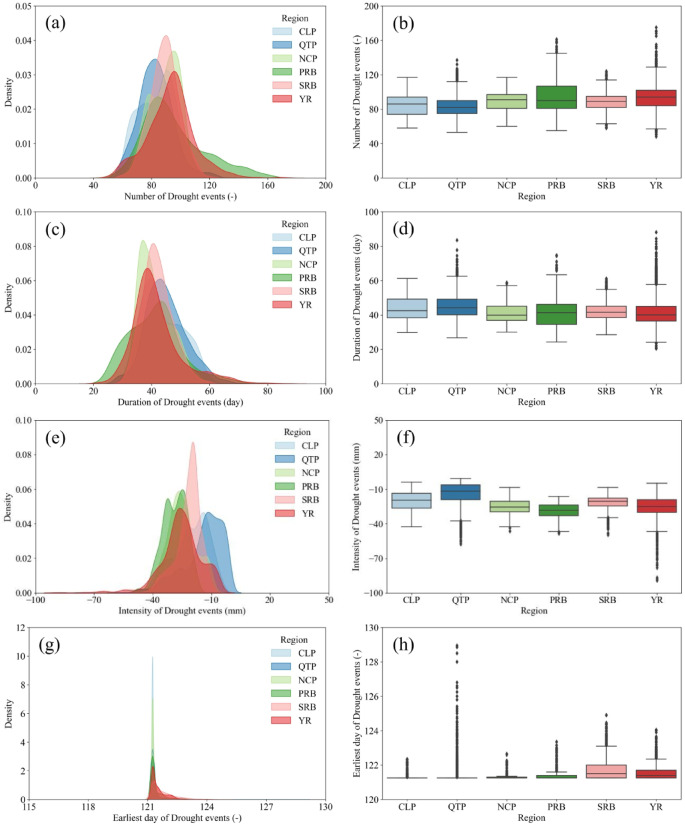
Probability distributions and magnitude statistics of total number of drought events (**a** vs. **b**), mean duration of drought events (**c** vs. **d**), mean intensity of drought events (**e** vs. **f**), and mean earliest day of the year for drought events (**g** vs. **h**) in six typical regions of China (2004–2023).

Figure 9 presents probability distributions. Drought number (Fig. 9a–b) is highest in PRB and YR (80–110 events), with right-skewed distributions, while the QTP records fewer events (70–90). Duration (Fig. 9c-d) is mostly 30–50 days, with the QTP peaking at ~ 50 days and the NCP exhibiting shorter durations and left-skewed distributions. Intensity (Fig. 9e–f) varies, with the PRB peaking between -30 and 20 mm and the QTP lowest at -10 to 10 mm, with a right-skewed distribution. Earliest occurrence (Fig. 9g–h) aligns with seasonal trends: droughts appear earlier in CLP and NCP (120th–125th days, late April) and later in QTP and SRB (~ 130th day, mid-May). The box plot indicates earlier medians, narrow distributions, and stable seasonality in CLP and NCP, while the QTP exhibits greater variability.

### Response of meteorological and hydrological variables to heatwave events

**Fig. 10:**
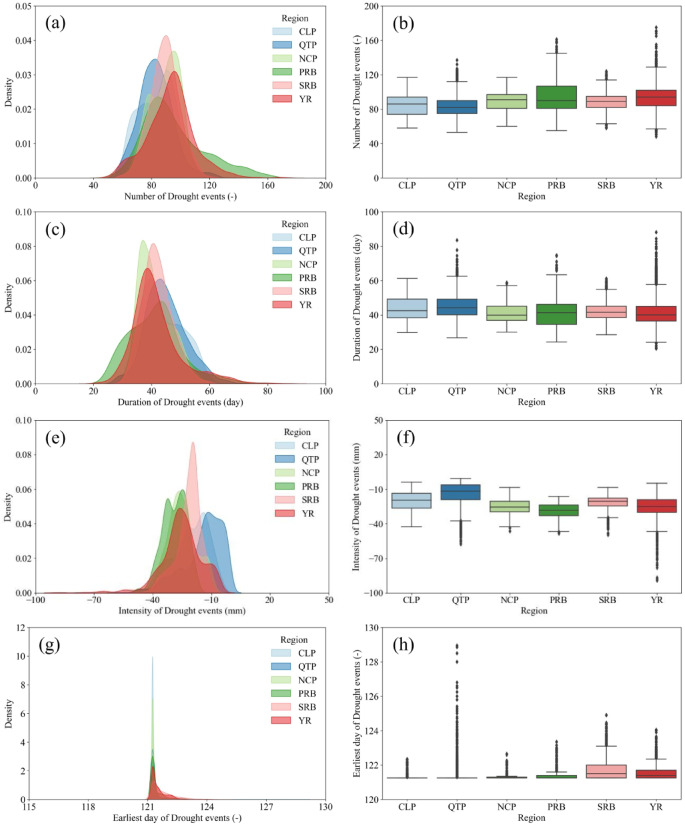
The average change in (**a**) Precipitation, (**b**) Temperature, (**c**) Relative Humidity, (**d**) Evapotranspiration, (**e**)Surface Runoff, (**f**) Root Zone Soil Moisture, (**g**) Ground Water Storage, and (**h**) Terrestrial Water Storage before and after heatwave events from 2004 to 2023 across the China (2004–2023). Elaborated with Spyder (Anaconda3) based on Python 3.9, https://www.spyder-ide.org/, https://www.python.org/

Figure 10 shows average climate and hydrological variable changes 3 days before and after heatwaves in China (2004–2023).Precipitation changes regionally (Fig. 10a). It rises in the Yangtze and Pearl River Deltas but drops in Xinjiang and western Inner Mongolia. Temperature generally increases post-heatwaves (Fig. 10b), markedly in the NCP, while changes are small in northern QTP high-altitude areas due to terrain and circulation. Relative humidity decreases in the NCP and increases in the Sichuan Basin (Fig. 10c), related to vapor sources and topography. Evapotranspiration varies spatially (Fig. 10d). It shows no change in many areas, rises in the vegetated Jianghan Plain, and is limited by water shortage in the Tarim Basin. Surface runoff increases in southeastern hilly areas after heatwaves (Fig. 10e), affecting the local hydrological cycle. RZSM decreases in parts of the NCP due to evapotranspiration and precipitation changes (Fig. 10f), but remains stable or increases in the YR Plain with irrigation or a good precipitation-evaporation balance. GWS may increase in the Northeast Plain and Yangtze Delta from runoff recharge (Fig. 10g), yet decreases in NCP cities from drought and over-exploitation. TWS changes are similar to GWS (Fig. 10h). It rises in the southeast coast from precipitation and low losses, and drops in the arid northwest due to low precipitation, high evaporation, and groundwater extraction, challenging sustainable development.

### Tracking the possible effects and drivers of meteorological and hydrological drought events

**Fig. 11:**
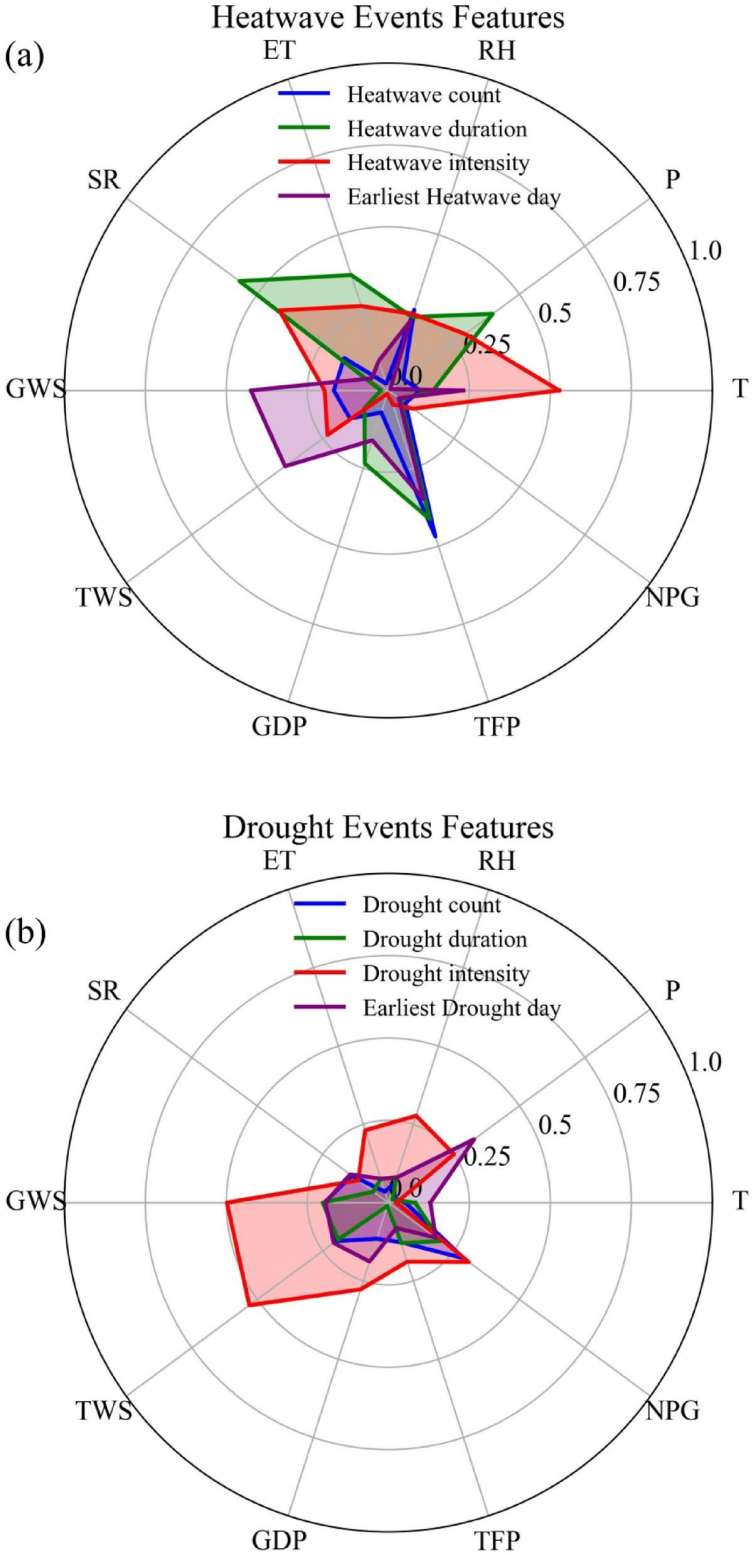
Correlation coefficient of natural and human factors including Relative Humidity (RH), precipitation (P), Temperature (T), Net Population Growth (NPG), Total Food Production (TFP), Gross Domestic Product (GDP), Terrestrial Water Storage (TWS), Ground Water Storage (GWS), Surface Runoff (SR), and Evapotranspiration (ET) on heatwave (**a**) and drought (**b**) events features such as count, duration, intensity, and earliest day.

Figure 11 presents correlation analyses between heatwave/drought characteristics and meteorological, hydrological, and socioeconomic drivers. For heatwaves (Fig. 11a), temperature exhibits the strongest association with intensity, while precipitation is closely linked to duration. Relative humidity uniformly influences all characteristics. Hydrological variables show runoff strongly correlates with duration and intensity, whereas GWS and TWS are most associated with the earliest onset. Socioeconomically, total food production correlates with event frequency, duration, and onset, and GDP correlates most strongly with duration; net population growth shows negligible correlations. Key drivers are thus food production with event number, runoff with duration, temperature with intensity, and GWS with onset timing. For droughts (Fig. 11b), net population growth correlates most strongly with event frequency, GWS with duration, and TWS with intensity. Rainfall most affects onset timing. Hydrologically, GWS and TWS show high correlations with intensity.

**Fig. 12:**
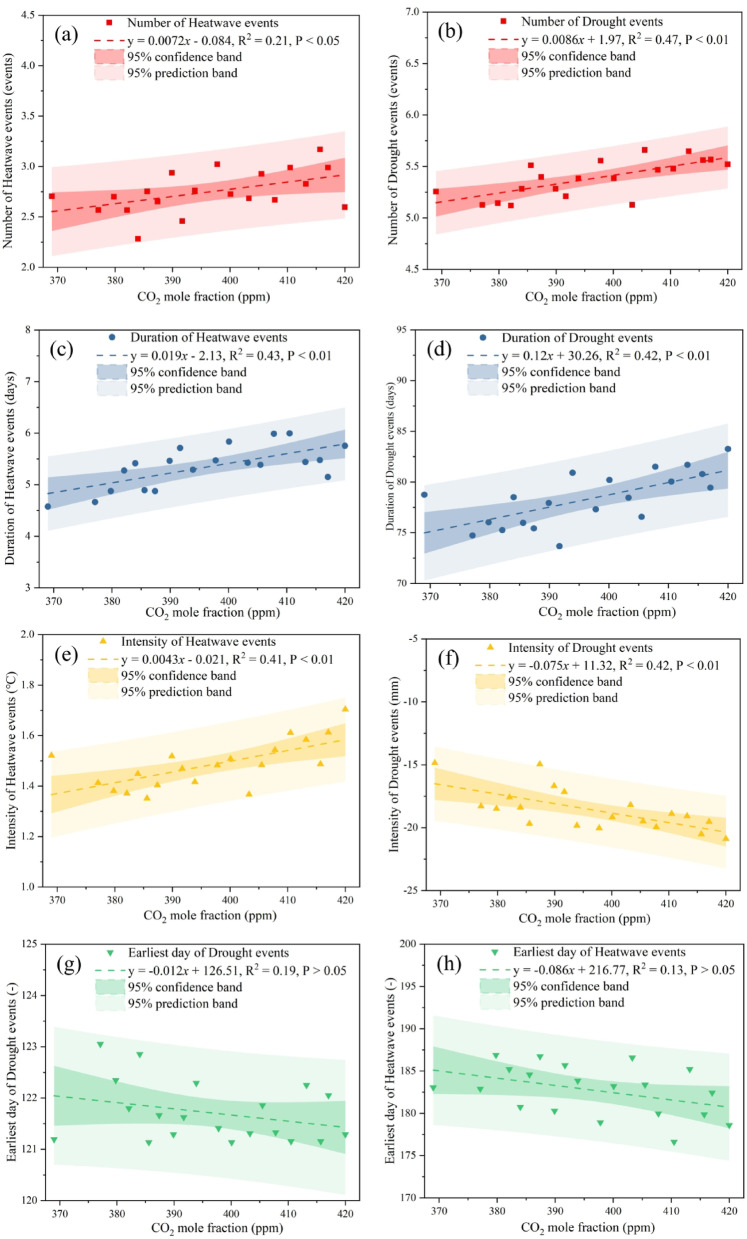
The exploratory association analysis of heatwave and drought event characteristics with global CO_2_ mole fraction over the period 2004 ~ 2023. Including mean number of drought events (**a** vs. **b**), duration of drought events (**c** vs. **d**), intensity of drought events (**e** vs. **f**), and earliest day of the year (**g** vs. **f**).

Figure 12 illustrates relationships between heatwave/drought metrics and global CO₂ mole fractions (2004–2023). Both event numbers increased with CO₂, particularly droughts (Fig. 12a–b). Durations intensified significantly (P < 0.01) by 0.019 and 0.12 days per ppm CO₂ for heatwaves and droughts, respectively (Fig. 12c–d). Intensity also increased (P < 0.01), with heatwaves warming 0.0043°C and drought RZSM decreasing 0.075 mm per ppm (Fig. 12e–f). Onset dates advanced non-significantly (Fig. 12g–h). Similar responses are observed for NH₄ and N₂O (Fig. S3), with significant increases in event frequency, duration, and intensity, and modest advances in onset for both hazard types.

## Discussion

### Spatio-temporal propagation and interaction mechanisms of heatwaves and droughts

This study reveals a significant increase and advancement in the frequency, intensity, duration, and timing of heatwave and drought events across six representative regions in China from 2004 to 2023. This trend aligns with the global pattern of intensifying extreme climate events under global warming^19,63^. Heatwaves exhibit notable spatial heterogeneity, particularly in the YR and NCP regions, reflecting the frequent occurrence of record-breaking heat events^23,45^. Potential drivers include the anomalous strengthening and persistence of the subtropical high, quasi-El Niño-like sea surface temperature distributions in the western Pacific, and intensified urban heat island effects due to urbanization^7,44^. The prolonged heatwaves in central regions further highlight the cumulative impact of urban warming. In contrast, while the absolute intensity of heatwaves in the QTP is relatively low, the rate of warming is substantial, with high-altitude ecosystems being more sensitive to temperature variations, posing significant ecological risks^42,43^. The spatiotemporal patterns of droughts are closely tied to precipitation distribution and human activity intensity. In densely populated and agriculturally developed areas, such as the NCP and YR downstream, droughts are frequent and prolonged, reflecting both natural precipitation variability and human-induced stress on water resources through intensive extraction and land-use changes^4,38^. This study also identifies a shift in drought intensity centers in the CLP and PRB, linked to long-term changes in the East Asian summer monsoon and groundwater over-exploitation^25,50^. Conversely, lower drought intensity in the northwest arid region is associated with limited human disturbance and more natural hydrological processes^25,46^.

Certain regions exhibit a distinctive pattern of low intensity but strong propagation. In the PRB, abundant annual precipitation and high background soil moisture limit the absolute intensity of heatwaves and droughts^25,64^. However, rapid urbanization, dense river networks, and intensive water resource management, such as inter-basin water transfers and reservoir operations, can effectively propagate hydrological and thermal anomalies, amplifying spatial transmission^65,66^. In contrast, the QTP has a high-altitude environment that suppresses extreme temperature and soil moisture intensity^42,67^, yet its fragile ecosystem is highly sensitive to disturbances. Even moderately intense events can trigger strong snow-soil-atmosphere feedbacks (e.g., reduced albedo, permafrost degradation), propagating disturbances across the plateau^26,68^. This highlights that event propagation is not solely driven by intensity but also significantly influenced by regional hydrometeorological sensitivity and human impacts. A central finding is the mutual reinforcement between heatwaves and droughts, forming a “composite dry-heat event.” In the NCP and Sichuan Basin, drought-induced reductions in RZSM amplify near-surface heat intensity by decreasing latent heat flux and increasing sensible heat flux^2,69^,conversely, intense heatwaves increase atmospheric evaporation, accelerating soil moisture depletion and exacerbating hydrological droughts^4,34^. This positive feedback loop, intensified by climate warming and human activities, leads to nonlinear amplification of disaster impacts^11,39^. In contrast, in the PRB, water resource availability buffers this coupling effect.

### Possible driving factors, environmental impacts, and socioeconomic effects of extreme events

Factorial analysis in this study provides a nuanced understanding of how various drivers influence heatwave and drought events. Heatwave characteristics, particularly intensity, show the strongest correlation with temperature, confirming that global warming is the primary driver of intensifying heatwaves^31^. Drought characteristics, however, are more closely linked to hydrological variables such as precipitation, GWS, and TWS, highlighting that droughts result from water balance deficits. Notably, net population growth correlates strongly with drought frequency, underscoring the role of rapid urbanization and socioeconomic development in increasing water demand, which exacerbates hydrological droughts, especially in water-stressed regions like the NCP^70^. This study also uses exploratory correlation analysis to examine the relationship between changes in heatwave and drought characteristics and the rising concentrations of key greenhouse gases (CO₂, CH₄, N₂O,Fig. 12). The results indicate a significant correlation between increased greenhouse gas concentrations and higher event frequency and intensity, as well as longer durations and earlier onset times. This aligns with the IPCC Sixth Assessment Report, which asserts that anthropogenic forcing is the primary driver of global-scale changes in extreme heat and precipitation, with future impacts projected to intensify under varying emission scenarios^63^. Greenhouse gases contribute to persistent heatwaves and widespread droughts by altering radiative forcing and atmospheric circulation patterns, such as causing high-pressure systems to stall^23,71^. It is important to note that this analysis represents an exploratory correlation to demonstrate significant synchronous trends and does not constitute causal attribution, as trends may be influenced by natural climate variability, other anthropogenic forcings, and feedback processes. The impact of heatwaves on regional water cycles exhibits spatial heterogeneity (Fig. 12. In the humid southeastern regions, increased precipitation and evapotranspiration may reflect enhanced convection but also accelerate water cycling; in northern arid regions, heatwaves coincide with reduced precipitation and constrained evapotranspiration, exacerbating water depletion and creating a vicious cycle of “high temperatures-low rainfall-water depletion”^22,40^. This hydrological pattern poses severe ecological risks, including vegetation water stress, altered species composition, reduced carbon sequestration, and heightened wildfire risk^72–74^.

Socioeconomic systems are highly vulnerable to extreme events. The strong correlation between total grain production and heatwave characteristics (Fig. 11a) highlights the risks to crop yields in China’s core agricultural regions, where high temperatures and water stress inhibit photosynthesis, accelerate growth, and lead to reduced yields or crop failure^16^. Droughts threaten drinking water security, industrial production, and hydropower generation, undermining water supply reliability and potentially triggering social instability through resource competition^14,28^. While the correlation between GDP and extreme events is weak, indicating short-term adaptive capabilities, frequent long-term events challenge economic resilience through supply chain disruptions, infrastructure damage, and rising health costs^13,15^. This study uses a 3-day buffer window to capture rapid hydrological responses to heatwaves, such as surface runoff, evapotranspiration, and shallow soil moisture, consistent with Hao et al.^4^. For slow-response variables like GWS and TWS, changes observed reflect short-term coupling with rapid processes, rather than long-term trends.

### Limitations and future research

This study, despite advancements in multi-source data fusion, spatiotemporal dynamics characterization, and multi-factor correlation analysis, has several limitations. While GLDAS data are validated by NOAA flux tower observations (R^2^≈0.97), uncertainties persist in hydrological variables from remote sensing and model inversion, particularly in complex regions like the QTP and areas with sparse stations, potentially affecting drought identification accuracy. The study primarily reveals statistical correlations, lacking detailed characterization of physical processes and biogeochemical feedbacks (e.g., vegetation-atmosphere feedback, soil moisture-temperature coupling). Socioeconomic analysis remains macro-level, with limited assessment of impacts such as heat-related illnesses, population migration, and economic inequalities. The disaster mitigation benefits of adaptation measures (e.g., irrigation efficiency, green space planning, early warning systems) were not quantified. The three-day buffer window fails to capture lag effects in slow-response variables like groundwater and total water storage. Additionally, linear trend extrapolation overlooks the nonlinear climate system and driving factor changes, and the greenhouse gas analysis remains an exploratory correlation, lacking rigorous attribution methods.

Future research should address current limitations and expand upon core findings in several key areas. First, integrating ground-based observations (e.g., soil moisture networks, GRACE/GRACE-FO satellites) with high-resolution reanalysis data will help reduce data biases. Additionally, using longer time windows, lag correlation analysis, or process models can capture multi-timescale hydrological responses. Structural equation modeling, causal discovery algorithms, or process-oriented land surface models can reveal the intrinsic causal relationships between extreme events and environmental variables. Second, coupling climate models with integrated assessment models for multi-scenario socioeconomic impact risk assessments is essential, prioritizing health impacts, population migration, and inequalities in economic loss distribution. Furthermore, quantifying the disaster mitigation benefits and feasibility of various adaptation strategies is critical for operational decision-making support. Regarding future risk projections, the 2024–2050 trend forecasts should be viewed as “empirical scenario extrapolations” based on statistical trends from 2004 to 2023, with the assumption that primary drivers will remain relatively stable in the near to medium term. Without mitigation, the risk of extreme heatwaves and droughts in China’s major river basins could increase by 1.14–1.33 times compared to current levels, underscoring the urgent need to meet Paris Agreement targets^63^. Adaptive capacity must also be enhanced through high-resolution climate-hydrological models, optimized water resources management, and multi-indicator early warning systems^17,75^. Lastly, multi-scale analysis, nonlinear modeling, and scenario analysis should strengthen the link between global forcing and regional impacts to identify vulnerabilities in water-scarce, densely populated areas.

## Conclusions

This study analyzed the dynamics, characteristics, impacts, and drivers of heatwaves and droughts from 2004 to 2023 across six major Chinese river basins, using GLDAS data validated by NOAA flux tower observations. The main conclusions are as follows:

1. Heatwave and drought events showed significant increases in frequency (0.019 vs. 0.022 events/yr), intensity (0.012 °C vs. 0.14 mm/yr), duration (0.039 vs. 0.4 days/yr), and earlier onset (0.23 vs. 0.045 days/yr). Empirical extrapolation based on these recent trends suggests that, should current conditions persist, their characteristics could potentially intensify by approximately 1.14–1.33 times, with onset advancing by 1.22–4.33 days by 2050. Greenhouse gas emissions are key drivers.
2. Spatially, the Yangtze River Basin faces frequent events, while the Qinghai-Tibet Plateau experiences fewer but more severe ones. The Songliao Basin shows bimodal heatwave peaks with high intensity; the Loess Plateau and Pearl River Basin exhibit frequent short events of varying intensity.
3. Post-heatwave temperature rises dominate northern regions, while southeastern areas show increases in precipitation, evapotranspiration, GWS, and RZSM.
4. Heatwave characteristics correlate strongly with temperature, runoff, and food production, while droughts align with precipitation, GWS, TWS, and population.

This study provides key insights into the spatiotemporal dynamics of compound heatwave and drought events across China. The identified historical trends and their potential near-future persistence underscore the urgency of emission reductions and adaptive management to mitigate escalating hydrological, agricultural, and societal risks. The findings offer critical scientific support for regional water resource management and ecological protection strategies in a changing climate.

## Supplementary Information

Below is the link to the electronic supplementary material.

Supplementary Material 1

## Data Availability

Data relevant to the analysis can be downloaded from topography datasets at https://earthengine.google.com; satellite meteorology data at https://disc.gsfc.nasa.gov/datasets/GLDAS_NOAH025_3H_2.1/summary; in-situ meteorology station data at https://www.ncei.noaa.gov/data/global-summary-of-the-day/archive/; satellite hydrology data at [https://disc.gsfc.nasa.gov/datasets/GLDAS_CLSM025_DA1_D_2.2/summary] socioeconomic data at https://www.gov.cn/; and greenhouse gas data at [https://wmo.int/publication-series/wmo-greenhouse-gas-bulletin-no-20.] For the processed derivative data generated in this study, if you need to request them, please contact the corresponding author, Liu Dongdong (Email: [ddliu@gzu.edu.cn](mailto:ddliu@gzu.edu.cn); [liudongdongcn@foxmail.com)](mailto:liudongdongcn@foxmail.com)) .
