# Supplementary material for: The characteristics and spatiotemporal evolution of heatwaves and droughts across six typical regions in China
Source: Sci Rep. 2026 Mar 18;16:14012. doi: 10.1038/s41598-026-43650-1 (PMC13133368; doi:10.1038/s41598-026-43650-1)
Supplement: Supplementary file 1 — Supplementary Material 1 [file 41598_2026_43650_MOESM1_ESM.docx]

# Supporting Information for

# The characteristics and spatiotemporal evolution of heatwaves and droughts across six typical regions in China

Yang Ya^1^, Liu Dongdong^1*^

^1^College of Resource and Environmental Engineering, Key Laboratory of Karst Georesources and Environment, Ministry of Education, Guizhou University, Guiyang, 550025, China

*Corresponding author. Liu Dongdong, Email: ddliu@gzu.edu.cn

Tel: 86+15185160228

## Validation of GLDAS data:

In this section, the performance of the GLDAS dataset is evaluated by comparing it with in-situ flux tower temperature data. High-quality, ground-based observations of temperature provided by the in-situ flux tower data serve as a reliable benchmark for assessing the accuracy of the GLDAS data. This validation approach is informed by the study of Hao et al. (2024).

### **Description of Figure S1:**

As shown in Fig. S1, a density scatter plot of 209542 data points was generated to visualize and compare the temperature data from both sources. In this plot, the color of each data point represents the density of observations in that region. The density plot shows that the data mainly concentrates between -6 degree Celsius to 24 degrees Celsius. The analysis of the density scatter plot reveals a strong linear relationship between the GLDAS dataset and the in-situ flux tower data, as indicated by the high coefficient of determination (R²) values of 0.97. However, some discrepancies were also observed, particularly in areas with lower temperature values. These discrepancies could be attributed to the differences in the spatial and temporal resolutions of the two datasets, as well as the limitations of the GLDAS model in capturing local-scale temperature variations. Overall, the density scatter plot analysis demonstrates that the GLDAS dataset performs well in representing the temperature variability observed in the in-situ flux tower data.

| 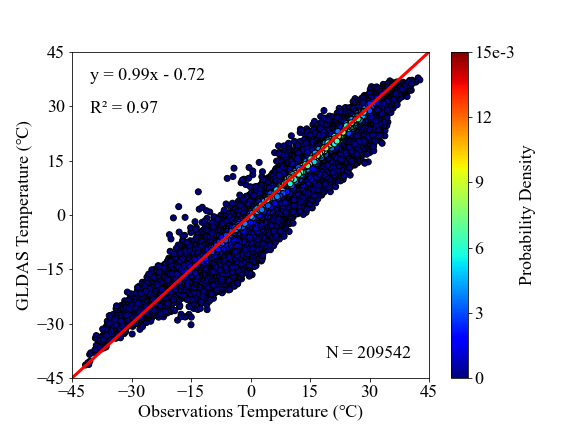 |
| --- |

### Fig. S1 Density scatter plot of GLDAS 2.2 temperature data vs. in-situ flux tower measurements.

### **Description of Figure S2:**

In the case of rainfall, the use of cumulative density functions (CDFs) for comparisons is due to the inherent variability and intermittent nature of precipitation events. Daily precipitation data often contain a significant number of zero values (no rainfall), which can affect the interpretation of statistical measures such as means or correlations. CDFs allow for a comprehensive analysis of the entire precipitation distribution, capturing the probabilities of various rainfall intensities (Dinku et al., 2008). Fig. S2 presented herein illustrate the comparison of two datasets using CDFs. The visual comparison of CDFs on the same graph allows for the identification of similarities or differences in the underlying probability distributions of the datasets. In general, overlapping or closely shaped CDFs suggest that the two datasets may have similar or the same distribution, while significantly different CDFs indicate that the datasets may have different distributions. Upon analysis, it was found that the sample sizes of the sites were concentrated between 6920-7295.The CDFs for KARAMAY, CH and TAZHONG, CH show a perfect fit, with the two lines overlapping almost exactly. However, it should be noted that not the larger the sample size, the more reliable the results. For example, KARAMAY, CH has a sample size of 6920, but his CDF is much closer than that of TULIHE, CH, which has a sample size of 7295, suggesting that there may be other potential biases in comparing CDFs. For example, the spatial and temporal variability of rainfall data may lead to errors when comparing datasets from different regions and time periods. In addition, localized weather patterns (e.g., thunderstorms or microclimates) can introduce variations in rainfall distributions that may not be accurately captured by weather models, leading to incorrect predictions of weather variables. These factors may lead to slight biases in the CDF.

| 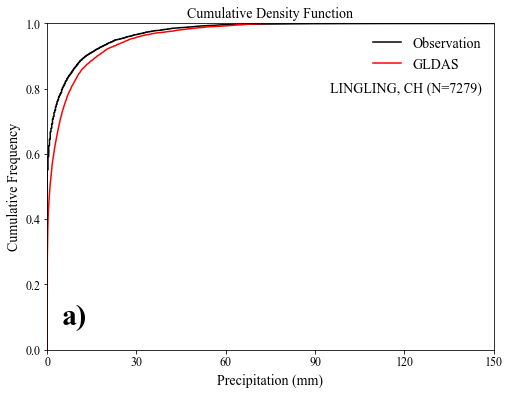 | 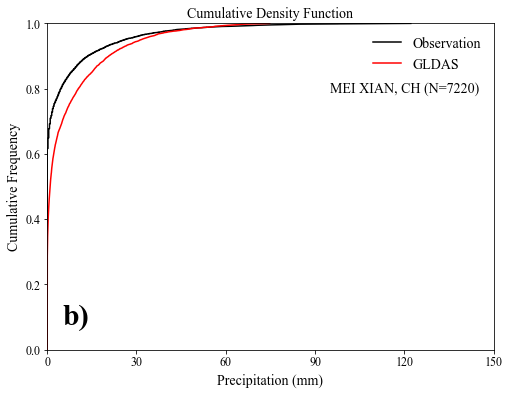 | 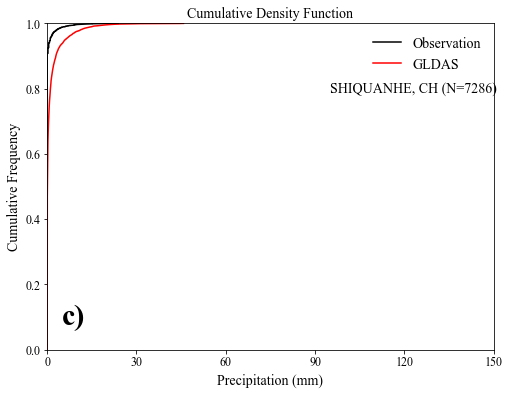 | 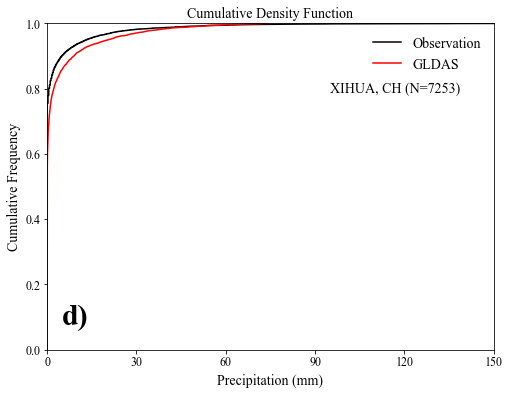 | 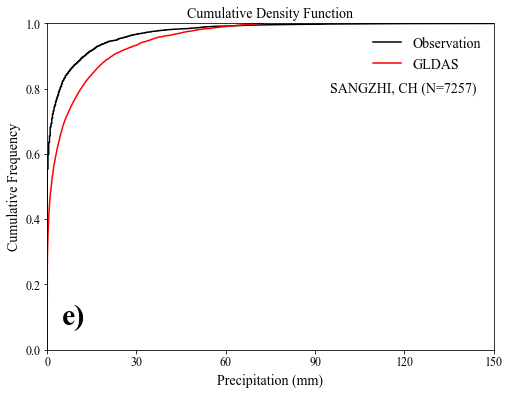 |
| --- | --- | --- | --- | --- |
| 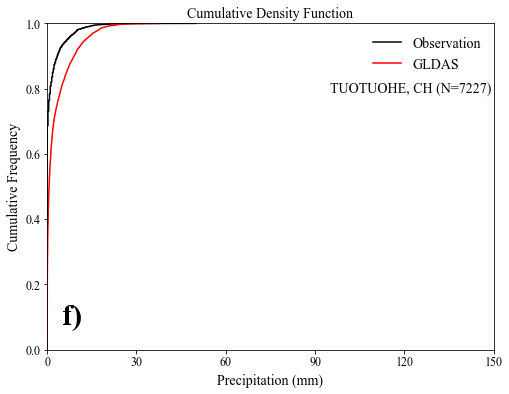 | 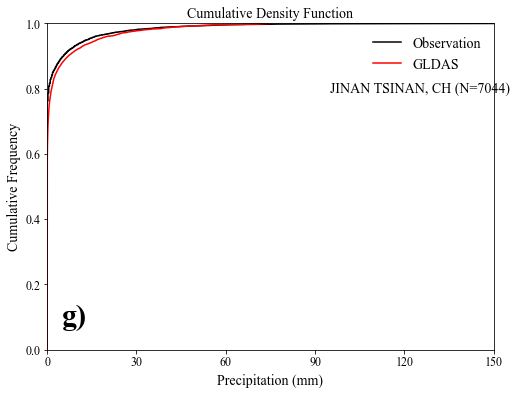 | 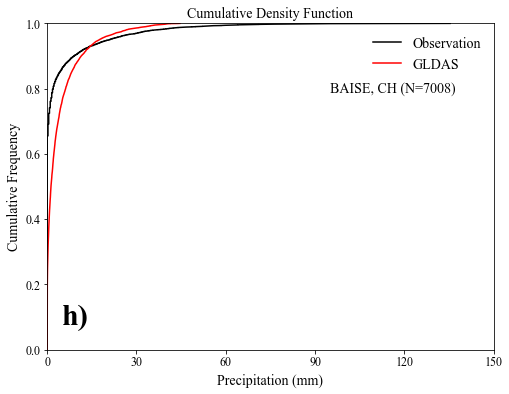 | 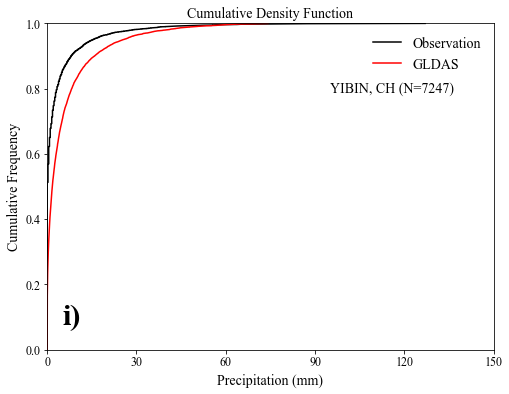 | 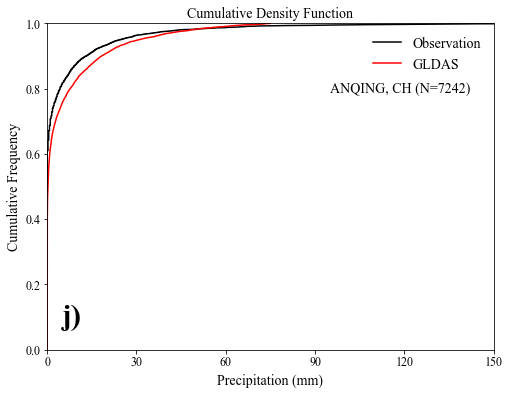 |
| 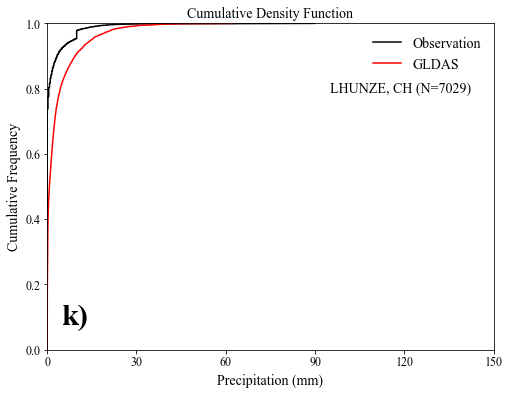 | 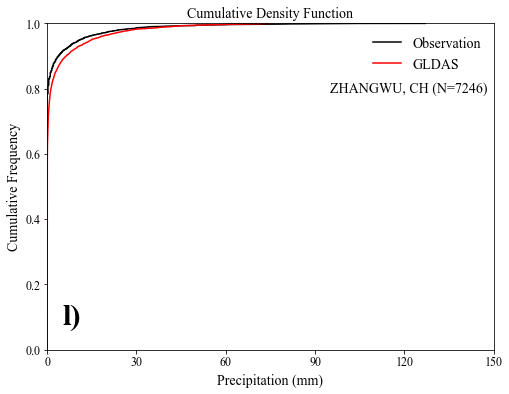 | 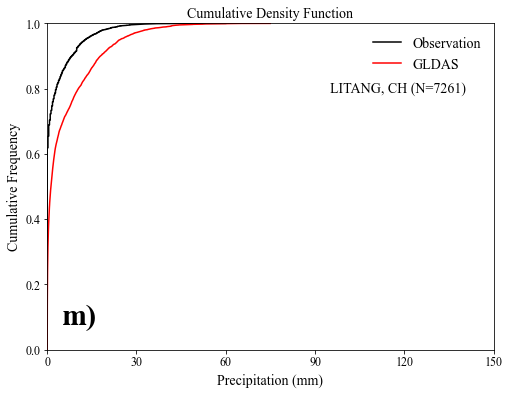 | 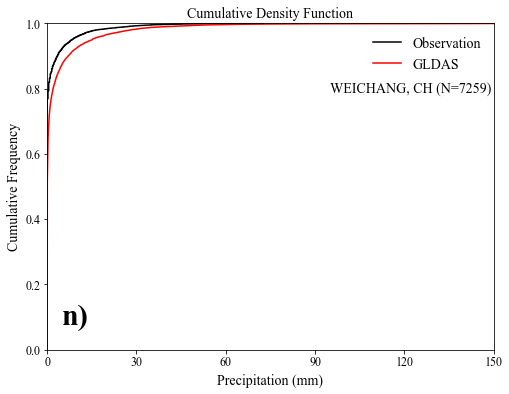 | 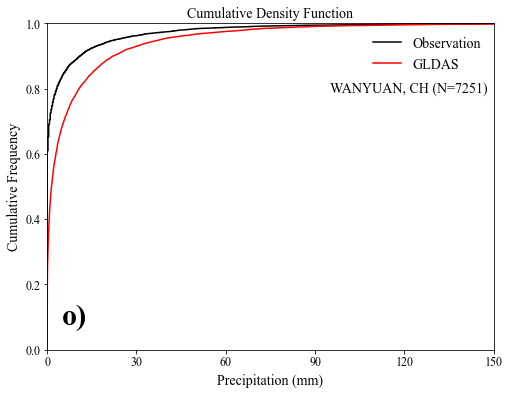 |
| 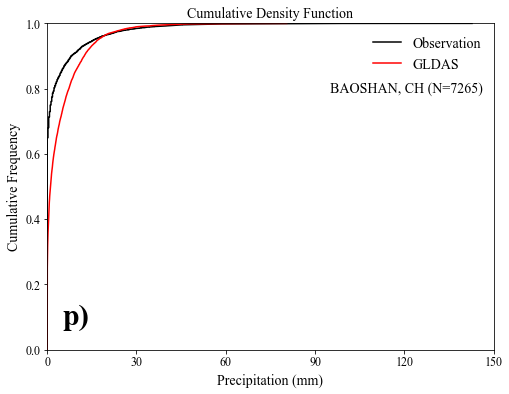 | 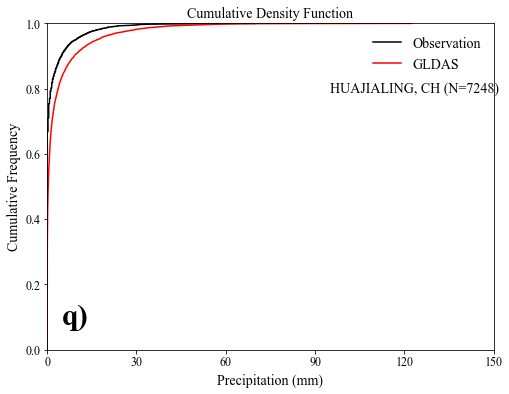 | 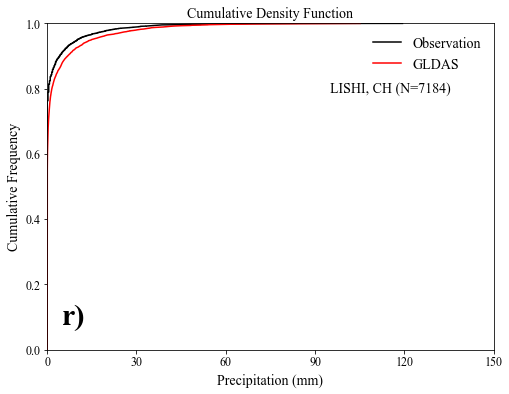 | 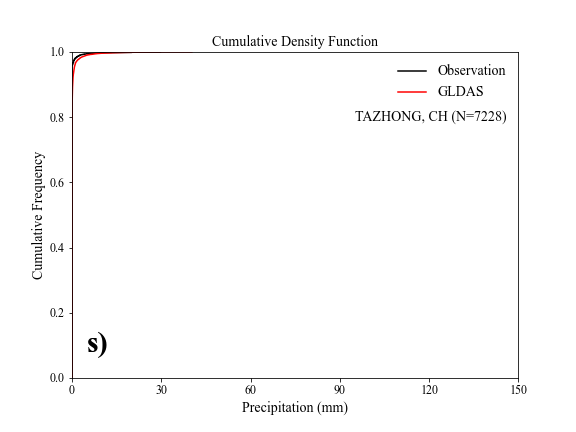 | 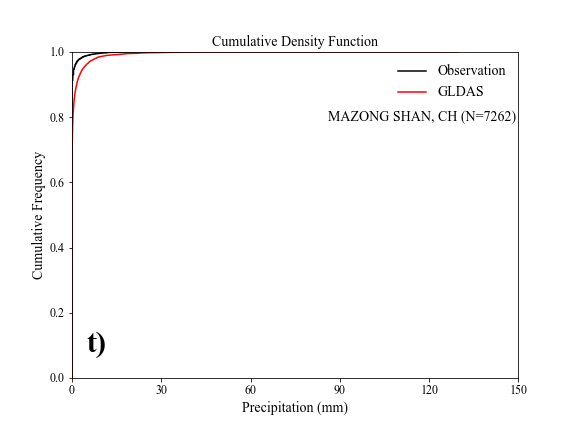 |
| 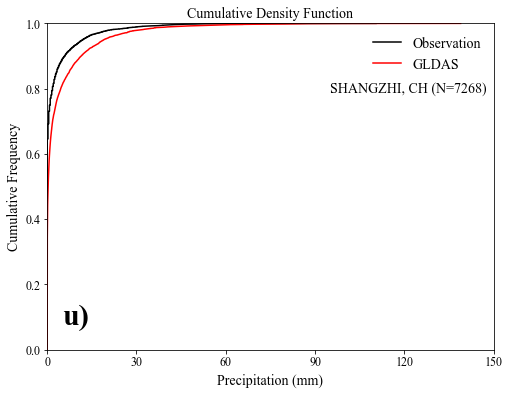 | 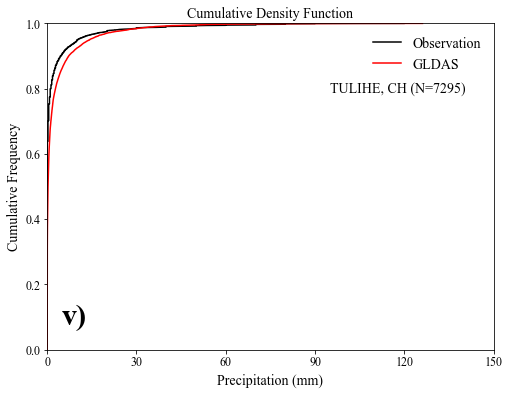 | 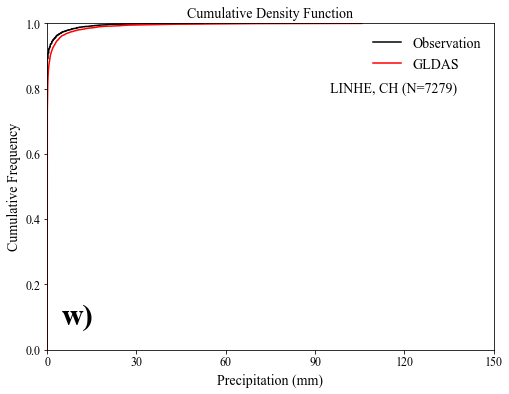 | 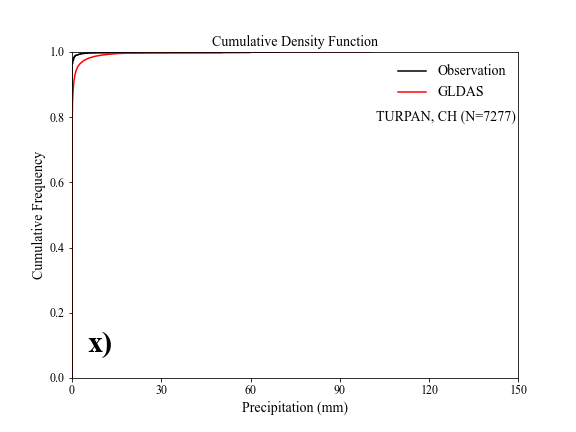 | 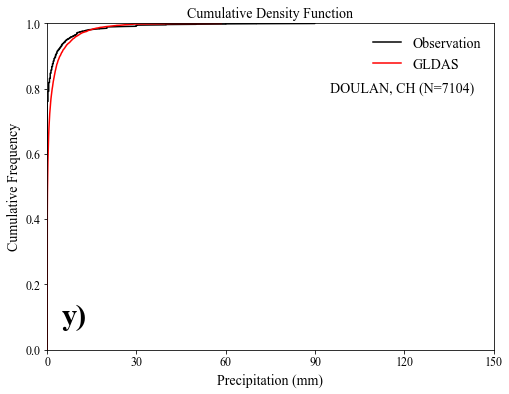 |
| 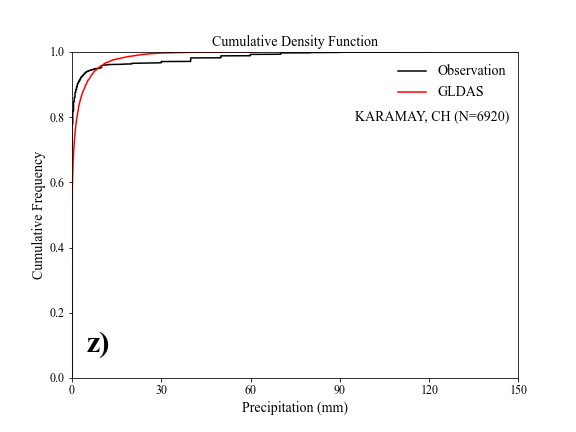 | 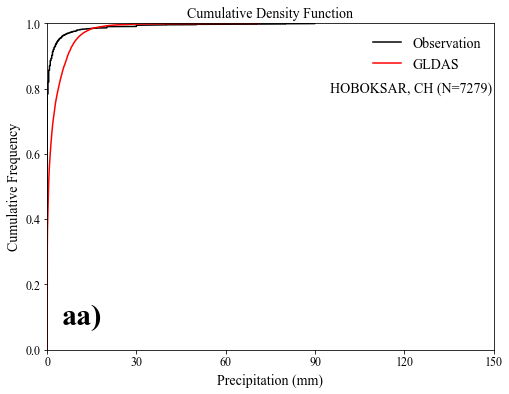 | 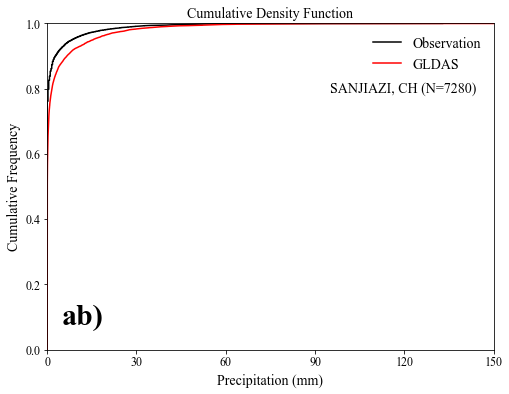 | 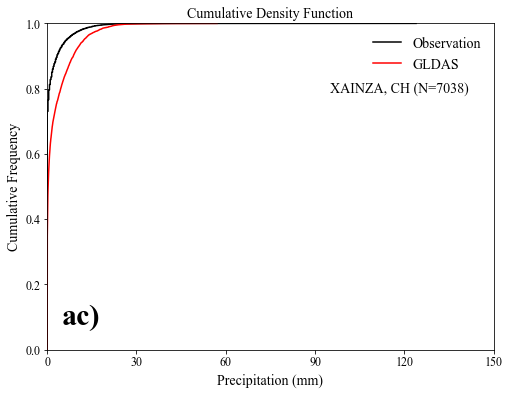 |  |

### **Fig. S2** Comparison of cumulative density functions (CDFs) for daily observed rainfall data and GLDAS data.

## Supplement of figure:

| 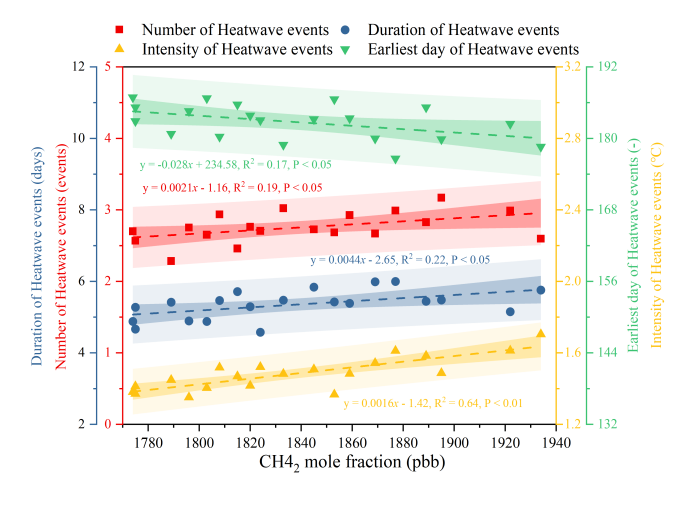  **A)** | 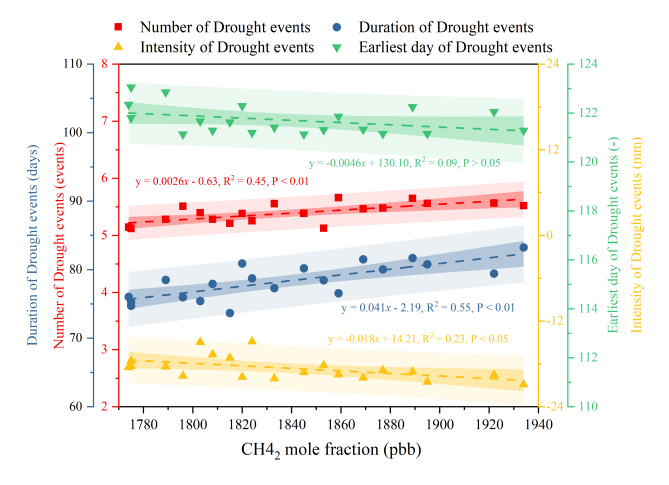  **a)** |
| --- | --- |
| 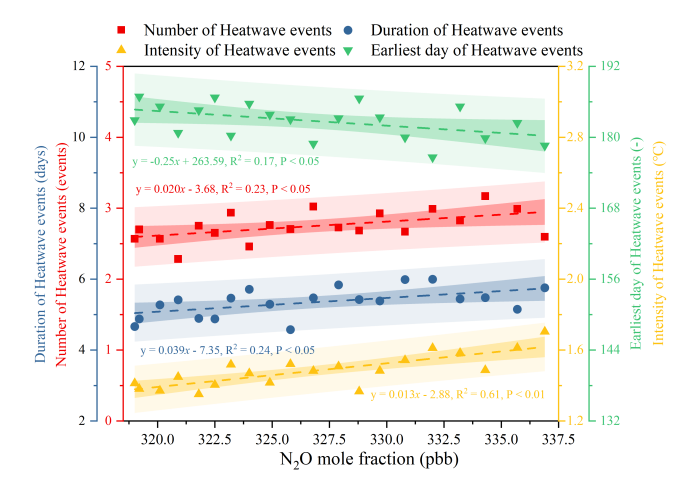  **B)** | 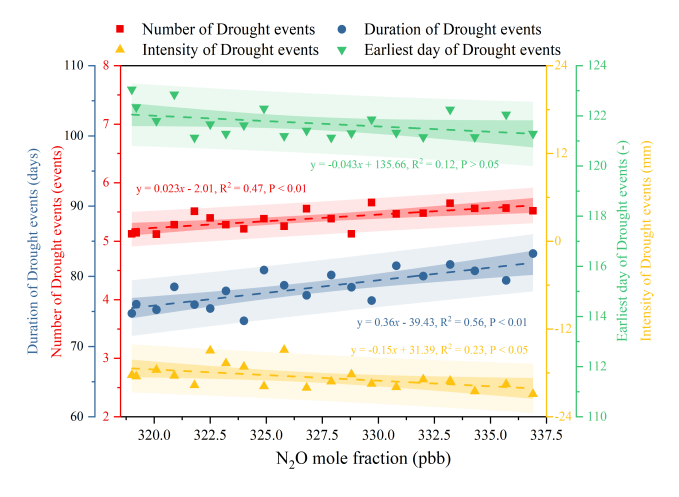  **b)** |

### **Fig. S3** The relationship between heatwave and drought event characteristics and global NH_4_ (A vs. a) and N_2_O (B vs. b) mole fraction over the period 2004 ~2023.

## Reference

Dinku, T., Connor, S. J., Ceccato, P. N., Ropelewski, C. F., 2008. Comparison of global gridded precipitation products over a mountainous region of Africa. <https://doi.org/10.1002/joc.1669>.

Hao, Y., Mao, J., Jin, M., et al., 2024. Evaluating the effects of heatwave events on hydrological processes in the contiguous United States (2003–2022). J. Hydrol. 637, 131368. [https://doi.org/10.1016/j.jhydrol.2024.131368](https://doi.org/10.1016/j.jhydrol.2024.131368).
